# Molecular function-guided unsupervised similarity-based enrichment for low-data virtual screening

**DOI:** 10.1186/s13321-026-01326-y

**Published:** 2026-11-06

**Authors:** Alexander Hagg, Dirk Reith, Matthias Preller, Karl N. Kirschner

**Affiliations:** 1 Department of Engineering and Communication, University of Applied Sciences Bonn-Rhein-Sieg, Grantham-Allee 20, 53757 Sankt Augustin, Germany; 2 Institute of Technology, Resource and Energy-efficient Engineering, University of Applied Sciences Bonn-Rhein-Sieg, Grantham-Allee 20, 53757 Sankt Augustin, Germany; 3 Fraunhofer Institute for Algorithms and Scientific Computing, Schloss Birlinghoven, 53754 Sankt Augustin, Germany; 4 Department of Natural Sciences, University of Applied Sciences Bonn-Rhein-Sieg, Von-Liebig-Straße 20, 53359 Rheinbach, Germany; 5 Institute for Functional Gene Analytics, University of Applied Sciences Bonn-Rhein-Sieg, Von-Liebig-Straße 20, 53359 Rheinbach, Germany; 6 Department of Computer Science, University of Applied Sciences Bonn-Rhein-Sieg, Grantham-Allee 20, 53757 Sankt Augustin, Germany

**Keywords:** Virtual screening, Unsupervised learning, Manifold learning, Molecular descriptors, Low-data regimes, Cheminformatics

## Abstract

Identifying bioactive compounds for orphan or understudied protein targets remains a significant hurdle in drug discovery due to the lack of sufficient structure-activity relationship (SAR) data. We present MolFuSE (Molecular Function-guided Similarity Explorer), an unsupervised, projection-based enrichment framework designed for low-data virtual screening and ligand repurposing. MolFuSE leverages manifold learning—specifically uniform manifold approximation and projection (UMAP)—to create low-dimensional similarity spaces based on a reference set of ligands known to bind proteins with the same molecular function as the target. Through systematic evaluation across nine protein targets and their functional classes, we demonstrate that UMAP embeddings of Mordred 2D descriptors act as a strong “potency filter”, significantly outperforming traditional topological fingerprints in the early enrichment of high-potency inhibitors (<100 nM). While extended-connectivity fingerprints (ECFP4) fingerprints achieve superior global ranking (receiver operating characteristic (ROC)-area under the curve (AUC) 0.961 vs. 0.863), the descriptor-based approach provides more robust early recognition (enrichment factor at 1% (EF@1%)) when the reference set itself is small. Crucially, we identify a critical point in data scaling: performance peaks at a reference set size of approximately 10,000 compounds and degrades for larger sets due to descriptor-space dilution from structurally diverse weak binders. MolFuSE provides a computationally efficient, interpretable tool for prioritizing high-affinity candidates when target-specific data is unavailable.

**Supplementary Information:**

The online version contains supplementary material available at https://doi.org/10.1186/s13321-026-01326-y.

## Scientific contribution

We introduce a data-pooling strategy for low-data virtual screening and ligand repurposing that uses molecular-function annotations to aggregate “pseudo-active” reference sets, bypassing the need for target-specific ligands or structural data. Evaluating this framework across eight diverse protein families, we show that non-linear UMAP projections of physicochemical descriptors act as a robust potency filter, preferentially prioritizing high-affinity binders (< 100 nM) in early enrichment compared to traditional topological fingerprints. We provide evidence of a critical scaling threshold where reference sets exceeding approximately 10,000 diverse compounds dilute the descriptor space, establishing a practical blueprint for unsupervised screening across distinct target classes.

**Supplementary Information:**

The online version contains supplementary material available at https://doi.org/10.1186/s13321-026-01326-y.

## Introduction

Identifying novel bioactive compounds for emerging therapeutic targets remains a persistent challenge in early-stage drug discovery. For well-characterized protein targets with extensive SAR data, machine learning approaches have demonstrated remarkable success in virtual screening campaigns [6, 8, 15, 27, 56]. However, the majority of potential therapeutic targets—particularly those in understudied protein families or emerging disease pathways—lack the hundreds to thousands of known bioactive molecules required for robust supervised learning models  [46].

### The low-data virtual screening challenge

This low-data regime problem is particularly acute for novel targets like myosins, orphan G-protein-coupled receptors, [20, 54] and other undruggable proteins, [45, 61] for which experimental screening campaigns have yet to be conducted at scale. While large pharmaceutical companies may leverage proprietary databases, this data remains inaccessible to the broader research community. Furthermore, for truly novel targets—such as those identified in emerging pathogens or rare diseases—historical screening data may not exist. Traditional ligand-based virtual screening (LBVS) methods rely on the *similar property principle*: structurally similar compounds are likely to exhibit similar biological activities [21, 25, 59, 60] and proteins with the same function bind the same set of small molecules [52]. This principle underpins similarity searching, where known active compounds for a specific target protein serve as queries to identify novel candidates from large chemical libraries. Compounds are typically encoded as fingerprints (e.g., ECFP4 [48]) or physicochemical descriptors, enabling rapid comparison via metrics such as the Tanimoto coefficient [55]. However, if few or no ligands are known, then the pairwise similarity comparison becomes undefined. Structure-based virtual screening (SBVS) via molecular docking [1, 24, 30] offers an alternative pathway through the lock-and-key principle [10], but requires high-quality experimental structures—frequently unavailable for novel targets—and computationally expensive pose optimization. To reduce the SBVS computational cost, an LBVS prefiltering can be done. In practice, early retrieval performance in virtual screening is commonly quantified using the enrichment factor at 1% (EF@1%). Literature analyses indicate that traditional 2D LBVS methods typically yield median EF@1% values of approximately 16.3 (10%/90% percentiles [12.7, 21.7]), whereas 3D shape-based methods can achieve higher median values near 27.8 (10%/90% percentiles [27.6, 28.0]) on custom benchmarking sets [50]. Crucially, these metrics are not directly comparable across studies due to differing target compositions and baseline difficulties. For instance, structure-based molecular docking protocols evaluated on distinct, potentially more challenging benchmarks like the DUD-E dataset show mean EF@1% values of approximately 8.09 for standard tools like AutoDock Vina [40], though multi-reference implementations can yield higher rates. These historical metrics establish a performance context for low-data screening frameworks.

### Manifold learning from related proteins

Homology-based similarity search has been used to identify ligands for a reference target when no initial ligands are known, by screening compounds that bind to homologous targets [49]. The authors introduce a descriptor based on four molecular features and calculate the molecular similarity between the candidate compound and the reference set by measuring the distance to its nearest neighbor(s). While the method yields high-quality rankings, the linear similarity space is entirely determined by the four features. Consequently, it may not perform well in highly diverse molecular spaces.

Following developments in chemogenomics, which have identified that certain molecular families can bind similar proteins, cross-target supervised approaches have been developed to screen the chemical space against entire protein families when known ligands for a target protein are absent [19]. When comparing binding affinity predictions using family-specific versus generic protein–ligand complexes, the family-specific models consistently yielded better predictions [58].

Supervised approaches, as described above, require datasets with affinity annotations, whereas unsupervised approaches can benefit from richer datasets. Herein, we exploit unsupervised learning methods, specifically *manifold learning*, from functionally related proteins. We define the set of functionally related proteins as the *functional class* of a target protein. For example, if a novel transferase target has no known inhibitor, one might leverage the 100,000+ known transferase inhibitors from associated target proteins to guide the screening. To our knowledge, this “functional class based transfer” concept – defined as using collections of bioactive compounds, the reference set (RS), grouped by molecular function of their target protein (e.g., KW-0808 transferase proteins; KW-0560 oxidoreductase proteins), as annotated by UniProt,^1^ which itself is based on the Gene Ontology [13]—has not been extensively explored as a manifold learning method in virtual screening, particularly for non-linear approaches.

While using functional annotations to group proteins is an established concept [12], our approach specifically applies this concept to bootstrap virtual screening by enriching an ample, diverse purchasable chemical space (i.e., from ZINC [18]) with a targeted set of functionally relevant compounds. We hypothesize that a non-linear similarity space derived from the combined ZINC and functional class-specific datasets provides a more informative chemical landscape, enabling the identification of promising candidates, even for targets with few known ligands. In the search for ligands that show affinity to novel or orphan target proteins, we define a RS as the set containing ligands with known affinity values for proteins within the same functional class. Aggregating known ligands for all proteins associated with a target’s function yields a significantly larger and more diverse RS of “pseudo-active” compounds, guiding the search more effectively and covering a larger portion of the potentially active molecular space. However, to better characterize such a diverse set, a richer (i.e., higher-dimensional) descriptor set is needed, which in turn poses its own problem.

### Dimensionality reduction for chemical space navigation

High-dimensional descriptors (i.e., encoding spaces) suffer from the curse of dimensionality, where pairwise distances converge to the same magnitude, making comparisons meaningless [3, 5]. To mitigate this information loss, (non-)linear projections can be used to find an embedding space whose dimensionality is close to the number of dimensions necessary to fully describe the data (a.k.a., the “intrinsic dimensionality”) [4]. Since projection methods are lossy by themselves, and it is usually not possible to determine the intrinsic dimensionality, the embedding space dimensionality can be chosen to allow a certain amount of information loss. For example, in principal component analysis (PCA) the dimensionality reduction is achieved by retaining the number of principal components required to account for a predefined percentage of the total data.

Recent advances in projective dimensionality reduction, particularly UMAP [36], enable the construction of low-dimensional molecular similarity spaces that preserve both local neighborhoods and global structure. This technique has been successfully applied to chemical space visualization (i.e., chemography) [38, 41] and has very recently been benchmarked against other non-linear projection methods for that purpose [42].

Unlike linear techniques such as PCA, which often struggle to preserve non-linear local similarity relationships [33, 42], or alternative non-linear methods like t-SNE, which do not preserve global spatial relationships and are computationally difficult to scale to large datasets [26], UMAP balances local and global structures while facilitating out-of-sample mapping without model retraining [2]. While modern deep learning representation methods—such as Graph Neural Networks and pre-trained Transformer models like ChemBERTa or MolBERT—capture highly complex chemical features [7, 9, 32], they typically require substantial computational resources and many training examples for fine-tuning, alongside offering limited direct interpretability compared to explicit distance-based metric spaces. This makes unsupervised manifold learning an attractive alternative to navigate these limitations. However, whether embeddings trained on a generic RS can effectively prioritize high-potency compounds for specific low-data targets remains an open question. In this work, we investigate this problem using MolFuSE.

### MolFuSE framework and contributions

MolFuSE (Molecular Function-guided Similarity Explorer) is a projection-based enrichment framework designed explicitly for low-data virtual screening scenarios. MolFuSE operates under the following three core design principles:

1. Projection-only strategy: Dimensionality reduction models are fit exclusively on the large RS plus decoy libraries, ensuring that held-out target actives do not influence the learned similarity space—mimicking realistic prospective screening conditions.
2. Exact nearest-neighbor scoring: Compounds are ranked according to their minimum Euclidean distance to the RS within the manifold (i.e., the embedded space). This provides interpretable retrieval paths and facilitates post-hoc analysis of enrichment drivers.
3. Potency-stratified evaluation: Performance is assessed separately for high-potency (affinity <100 nM), medium-potency (100–1000 nM), and weak (1000–10000 nM) actives, revealing method-specific biases toward different potency regimes.

Through four systematic experimental phases, we explore MolFuSE’s performance using several criteria: (1) hyperparameter optimization of dimensionality reduction methods (i.e., PCA vs. UMAP) and molecular representations (i.e., 1,611 Mordred 2D descriptors vs. 2048-bit ECFP4 fingerprints), (2) sensitivity to RS affinity cutoffs before scoring (i.e., filtering RS by affinity thresholds), (3) robustness to RS size (i.e., random subsampling from 10 to 100,000+ compounds with full model retraining), and (4) cross-target generalization (i.e., across eight target proteins from various functional classes with varying natural RS sizes).

Our findings reveal that UMAP embeddings of Mordred descriptors achieve good enrichment for high-potency ligands, substantially outperforming both traditional fingerprint methods and literature-reported 3D docking benchmarks. We demonstrate that adequate screening is achievable with an RS as small as 1,000 compounds, and identify a previously unreported critical point where an extensive RS degrades performance–likely due to descriptor space dilution from structurally diverse low-affinity binders. These results establish MolFuSE as a practical, computationally efficient tool for hit discovery in low-data regimes, with implications for orphan-target prioritization and early-stage drug-discovery workflows. In other words, MolFuSE is a ligand-repurposing tool that projects compounds with known activity against a particular protein class onto low-data targets, thereby suggesting candidates for experimental exploration.

## Methods

Our experimental framework relies on the interplay between three distinct compound sets: (1) the RS, comprising known binders to a functional class of proteins; (2) the Target Actives Set, a held-out collection of ligands with known affinity for a specific target protein (e.g., tyrosine-protein kinase ABL1) that is used for evaluation; and (3) the Decoy Set, a large library of presumed non-binders used to challenge the retrieval capability. The transferase functional class is used to illustrate and evaluate MolFuSE.

### Datasets

#### Target actives set: tyrosine transferase inhibitors

For Phase 1–3 experiments, we selected Tyrosine-Protein Kinase/Transferase ABL1 (CHEMBL1862) as the primary evaluation target since it has many known actives, making it a convenient target for this work. Known actives were retrieved from ChEMBL using the P00519 identifier, requiring affinity measurements between 1 and 10,000 nM and the *removal* of active compounds from the RS dataset, via canonical SMILES matching, to prevent data leakage between training and test sets. We also divided the active set into three potency tiers for evaluation:

- High: affinity < 100 nM (N = 1,562 compounds, 46.9%)
- Medium: 100 nM ≤ affinity <1,000 nM (N = 729, 21.9%)
- Weak: 1,000 nM ≤ affinity ≤10,000 nM (N = 975, 29.3%)

By removing all known ligands of the target protein, we mimic a low-data environment where the models lack any prior binding information. We did not artificially prune structurally similar analogs between RS and active set because our objective was specifically to evaluate whether the manifold could leverage information from related binders in the same functional class to enrich the target’s actives.

#### Reference set: transferase inhibitors

For the Tyrosine-Protein Kinase/Transferase ABL1 (CHEMBL1862) target protein, the UniProt functional class KW-0808 Transferase was selected. The target-specific RS was constructed from ChEMBL (version 34) [14] by querying all compounds with reported bioactivity with human transferase target proteins. Compounds were required to have standard binding affinity measurements (EC$_{50}$, IC$_{50}$, K$_i$, or K$_d$) in nanomolar units, and valid SMILES strings with successful RDKit parsing.

For compounds with multiple measurements against the same or different transferase targets, we applied median affinity deduplication: the median affinity across all assays was computed and assigned as the compound’s representative value. This approach provides robustness against multi-assay variability and batch effects common in large-scale screening databases [11]. In an uncharacterized or orphan target setting, the true binding site topology is unknown. If a compound shows strong affinity against one specific target protein but weak affinity across five other proteins in the same functional class, recording its “best” value would mischaracterize it as a universally potent binder for that functional class. The final RS contained approximately 191,000 unique transferase inhibitors with median affinity values ranging from sub-nanomolar to >10 μM.

For Phase 4’s cross-target validation, we selected eight additional targets spanning diverse structural families and RS sizes (43,000–425,000 compounds): ABL2, EGFR, ERBB2, SRC, LCK, YES1, FYN and LYN.

#### Decoy set: ZINC database

To simulate realistic screening conditions, we incorporated a decoy library from the ZINC database [18], representing commercially available “screening-like” compounds with no known transferase activity. After removing overlap with the RS or target actives (by canonical SMILES), the final decoy set contained 1,293,566 compounds. The decoy-to-active ratio is 58:1 with strict SMILES-based deduplication.

### Molecular representations

To capture diverse aspects of chemical similarity, we utilized two distinct encoding strategies—explicit physicochemical descriptors (Mordred) to capture molecular properties, and topological fingerprints (ECFP4) to capture substructural features.

#### Mordred 2D descriptors

A total of 1,611 two-dimensional (2D) molecular descriptors were utilized in this study, calculated via the Mordred library [39]. These descriptors are summarized by category, descriptor family, and weighting properties in the supporting information. For a full listing, we refer to the code repository (see Sect. Limitations and future directions).

Preprocessing the resulting data consisted of the following steps:

1. Zero-variance filtering: Descriptors with variance $< 10^{-12}$ (effectively constant across all compounds), or those where no descriptor values were calculable, were removed, yielding approximately 1,611 informative descriptors.
2. Median imputation: Missing descriptor values, around 5.95% on average per descriptor (e.g., from SMILES parsing failures or undefined mathematical operations), were replaced with the median descriptor value computed across the RS and decoy set.
3. Standardization: The remaining descriptors were z-score normalized, fit exclusively on the combined RS and decoy set. Held-out target actives were transformed using the same scaler parameters, ensuring no data leakage.

StandardScaler was applied *only* to Mordred descriptors. Fingerprint representations (see below) were used directly without scaling.

#### ECFP4 fingerprints

For comparison, we generated 2048-bit Extended Connectivity Fingerprints with radius 2 (ECFP4) [28, 48]. Fingerprints were stored as binary vectors (0/1 for absence/presence) and used without preprocessing.

### Dimensionality reduction algorithms

PCA and UMAP models were fit on the combined RS and decoy set (N = 1,484,566 compounds: 191,000 RS + 1,293,566 decoys). Held-out target actives were projected into the learned embedding space using the respective transform method, ensuring strict separation between training and test sets. Both methods were used to project the dataset into 2D, 5D, 10D and 20D embedding spaces. Both methods used either the binary fingerprints directly or z-score-normalized Mordred descriptors.

The following key UMAP hyperparameters were optimized in Phase 1:

- n_neighbors ∈ {10, 20, 50, 100, 500}: Controls local vs. global structure preservation. Lower values emphasize tight local clusters; higher values capture broader manifold geometry.
- min_dist ∈ {0.0, 0.01, 0.1}: Minimum distance between embedded points. Lower values allow tighter clusters; higher values produce more dispersed embeddings.
- n_components ∈ {2, 5, 10, 20}: Dimensionality of the target embedding space.
- metric: Distance metric for manifold construction. Employed were the euclidean metric for Mordred descriptors, and jaccard for ECFP4 fingerprints due to their binary bit-vector nature.
- random_state: Set to None to enable parallel processing across independent replicates and for variance assessment.

To ensure reproducibility, all UMAP runs used five independent replicates, while the deterministic PCA runs are reported as single values. The RS subsampling (Phase 3) used five independent random samples per size, and the cross-target validation (Phase 4) used five replicates per target. Performance metrics are reported as mean ± standard deviation across replicates.

### Scoring and ranking

After embedding, all compounds (i.e., RS, decoys, and held-out actives) were represented as points in the low-dimensional space. For each held-out active compound, we computed its ranking score as:

1 $$\begin{aligned} \text {score}(x_{\text {active}}) = -\min _{x_{\text {RS}} \in \text {RS}} d(x_{\text {active}}, x_{\text {RS}}) \end{aligned}$$

where $d(x_{\text {active}}, x_{\text {RS}})$ is the Euclidean distance in the embedded space. The negative sign ensures that compounds *closer* to the RS receive *higher* scores. This exact 1-nearest-neighbor (1-NN) approach was implemented using a kd-tree for computational efficiency with Euclidean metrics. The evaluation set compounds (i.e., target actives and decoys) were ranked by descending score, and enrichment metrics were subsequently computed.

### Computational implementation

All preprocessing, dimensionality reduction, and scoring were implemented in Python 3.12. SMILES parsing, ECFP4 fingerprint generation [48], EF@1%, ROC-AUC, and Boltzmann-enhanced discrimination of ROC (BEDROC) were computed using RDKit 2023.03.1 [29]. Mordred community edition 2.0.6 [39] was used for 2D descriptor computation. Data manipulation and numerical computation were done using Pandas [37, 43] and NumPy [16]. The scikit-learn 1.3.0 package [44] was used to compute PCA, StandardScaler, KDTree, imputation and zero-variance filtering. The fast umap-learn 0.5.3 library [17, 36] was used for computing UMAP.

### Experimental design

#### Evaluation metrics

While global metrics like ROC-AUC and precision-recall (PR)-AUC assess overall ranking quality, they can fail to capture “early enrichment”—the ability to find true actives in the top *x*% of predictions [23, 31, 35, 50, 57]. A high global AUC does not guarantee that actives are positioned early enough for experimental validation [57]. Consequently, we utilize EF@1% and BEDROC to specifically evaluate early enrichment. The EF@1% quantifies how many true actives appear in the top 1% of ranked compounds relative to random selection [57].

2 $$\begin{aligned} EF_{1\%} = \frac{\left( n_{\text {active in top 1\%}} / n_{\text {top 1\%}} \right) }{\left( n_{\text {active}} / N_{\text {total}} \right) } \end{aligned}$$

where $N_{\text {total}}$ represents the total number of compounds, and *n* represents the number of compounds in the respective subsets.

It was noted that EF@1% is not sensitive to ranking performance before and after the cutoff threshold [57]. The BEDROC metric was introduced to address this limitation by giving more weight to top-ranked compounds. Consequently, it provides a more robust measure of early enrichment that is less sensitive to a specific, arbitrary cutoff value. It is defined as:

3 $$\begin{aligned} \text {BEDROC}(\alpha ) = \frac{RIE(\alpha ) - RIE_{\text {min}}(\alpha )}{RIE_{\text {max}}(\alpha ) - RIE_{\text {min}}(\alpha )} \end{aligned}$$

where

4 $$\begin{aligned} RIE(\alpha ) & = \frac{\sum \nolimits _{i=1}^{n} e^{\frac{-\alpha r_i}{N_{\text {total}}}}}{\left\langle \sum \nolimits _{i=1}^{n} e^{\frac{-\alpha r_i}{N_{\text {total}}}} \right\rangle _{\text {random}}} \\ & = \frac{N_{\text {total}}}{n} \frac{\sum \nolimits _{i=1}^{n} e^{\frac{-\alpha r_i}{N_{\text {total}}}}}{\frac{1 - e^{-\alpha }}{e^{\frac{\alpha }{N_{\text {total}}}}-1}} \end{aligned}$$

5 $$\begin{aligned} & RIE_{\text {min}}(\alpha ) = \frac{N_{\text {total}}}{n} \frac{1 - e^{\frac{\alpha n}{{N_{\text {total}}}}}}{1 - e^{\alpha }} \end{aligned}$$

6 $$\begin{aligned} & RIE_{\text {max}}(\alpha ) = \frac{N_{\text {total}}}{n} \frac{1 - e^{\frac{-\alpha n}{{N_{\text {total}}}}}}{1 - e^{-\alpha }} \end{aligned}$$

The α is a tunable exponential weighting factor, $r_i$ is the rank of the $i^{th}$ active compound (from 1 to $N_{\text {total}}$), *n* is the number of active compounds, and $N_{\text {total}}$ is the total number of compounds within the dataset [47, 57]. The “early enrichment” factor α controls the weight given to the top-ranked compounds, tuning the metric towards quantifying early enrichment. A higher α places greater importance on compounds at the top of the ranked list. An α=20 is a common assignment where approximately 80% of the BEDROC score is determined by the top 8% of the ranked compounds. An $\alpha \approx 160$ is a highly stringent setting where 80% of the score is derived from just the top 1% of the ranked list.

#### Stratified potency tiers

To assess method-specific biases, we computed separate EF@1% values for high-potency (affinity < 100 nM), medium-potency (100–1000 nM), and weak (1000–10,000 nM) actives. This stratification reveals whether methods preferentially retrieve tight binders vs. weaker inhibitors – a critical distinction for hit-to-lead optimization.

#### Phase 1: hyperparameter optimization

We performed a systematic grid search across the following methods, representations, and hyperparameters:

- Dimensionality reduction methods: PCA and UMAP
- Molecular representations: Mordred (1,611 descriptors) and ECFP4 (2048-bit)
- UMAP hyperparameters: n_neighbors ∈ {10, 20, 50, 100, 500}, min_dist ∈ {0.0, 0.01, 0.1}, and n_components ∈ {2, 5, 10, 20}
- PCA hyperparameters: n_components ∈ {2, 5, 10, 20}

This yielded 515 total experimental runs. For each configuration, we computed overall EF@1% and potency-stratified EF@1% values. The optimal configuration was selected as the highest-performing method assessed using the overall EF@1%, with secondary analysis of high-potency-stratified performance.

#### Phase 2: affinity cutoff sensitivity (re-scoring only)

Using the optimal hyperparameters identified in Phase 1, we investigated if filtering the RS by affinity thresholds improves the enrichment. We re-scored the *same* Phase 1 embeddings (i.e., no model retraining) using RS filtered to include affinities a) ≤ 100 nM (i.e., high-potency compounds), b) ≤ 1,000 nM, c) ≤ 10,000 nM, and d) ≤ 100,000 nM. For each cutoff, exact 1-NN distances were recomputed to the filtered RS, and enrichment metrics were re-evaluated. This design isolates the effect of RS potency composition from embedding geometry.

#### Phase 3: reference set size ablation (full retraining)

To assess robustness to RS size, we performed the following systematic ablation study:

1. Random subsampling: The full RS was randomly subsampled to target sizes: 10, 100, 1,000, 10,000, and 100,000. Subsampling was performed *without replacement* from RS to test generalization capacity across diverse RS scales.
2. Model retraining: For each RS subsample, we restandardized the values and retrained the dimensionality reduction model (UMAP or PCA) from scratch using the optimal hyperparameters found in Phase 1 (i.e., UMAP 2D, n_neighbors=10, min_dist=0.0 for Mordred descriptors).
3. Evaluation: Held-out actives were projected into each newly trained embedding space and scored via exact 1-NN against the subsampled RS. The stratifying potency enrichment was then evaluated based on the optimal affinity cutoff (i.e., 100 nM).
4. Replicates: 5 independent random subsampling and retraining runs were performed per RS size to assess the variance.

This design tests the following hypothesis that larger RS provides better coverage of chemical space, improving retrieval, and, conversely, smaller set sizes degrade performance. The random subsampling approach (rather than curation or active learning) ensures that observed effects reflect *scale alone*, not content quality.

#### Phase 4: cross-target generalization

To validate generalizability beyond a single target, we applied the optimal hyperparameters from Phases 1–3 (i.e., see above plus 100 nM affinity cutoff) to eight additional targets with varying RS sizes: ABL2 (∼43,000), EGFR (∼86,000), ERBB2 (∼95,000), SRC (∼180,000), LCK (∼225,000), YES1 (∼280,000), FYN (∼340,000), and LYN (∼425,000 compounds). For each target, we:

1. constructed target-specific RS by excluding compounds with known activity against the held-out target (i.e., leakage prevention),
2. retrained the UMAP model on target-specific RS + ZINC decoys, and
3. evaluated potency-stratified enrichment on held-out actives (5 replicates per target).

We computed Spearman correlation between natural RS size and high-potency EF@1% to quantify the relationship between the RS scaling and the screening performance. This design tests whether the Phase 3 findings (optimal size 10,000, degradation at 100,000+) generalize across targets with naturally occurring RS sizes spanning 1–2 orders of magnitude.

#### Phase 5: ablation and decoy bias study

In this phase, we validated MolFuSE’s approach by conducting three control experiments that tested the necessity of dimensionality reduction and the magnitude of decoy bias.

#### Database bias negative control

To test if MolFuSE exhibits systematic bias toward ChEMBL-like compounds regardless of target specificity, we scored non-transferase actives against transferase-trained models. We selected seven diverse non-transferase target families from Phase 4 and scored their known actives using the respective transferase RS. Under the null hypothesis of no database bias, we expect random performance of EF@1% ≈ 1.0. Elevated enrichment would indicate the model preferentially ranks ChEMBL compounds over ZINC compounds, independent of biological relevance. However, critically, if this enrichment remains substantially lower than the target-specific performance, the databases are only *partially* separable with functional class specific-models increasing enrichment.

#### Raw descriptor ablation

To determine if UMAP dimensionality reduction is necessary, we scored candidates using the raw 1,611-dimensional Mordred descriptors with scaled Euclidean distance (1-NN). This control (35 replicates across seven Phase 4 target families) directly tests whether high-dimensional descriptor spaces provide adequate discrimination without the need for manifold learning. We compare raw descriptor performance against Phase 4 (descriptors + UMAP, averaged across all targets) using identical descriptors and scaling, isolating UMAP ’s contribution. The Phase 4 results represent an average behavior over diverse protein families, and thus provides a generalizable baseline rather than target-specific optimization.

#### Fingerprint ablation

To benchmark MolFuSE against established standards, we implemented raw ECFP4 fingerprint similarity using Tanimoto/Jaccard distance (1-NN, no UMAP). We compare against Phase 1 (ECFP4 + UMAP) across five replicates to quantify if UMAP’s learned metric provides meaningful improvement over established methods. This control also verifies that MolFuSE’s performance gains are not simply due to the use of ECFP4 representations.

The raw descriptor baseline and database bias control experiments used all eight Phase 4 target families (i.e., Antimicrobial, Antioxidant, Cytokine, Heparin-binding, Lyase, Motor Protein, Oxidoreductase, and Transferase; 35 total replicates) to assess generalizability across diverse protein families. The fingerprint baseline experiment (raw ECFP4 vs. Phase 1 ECFP4 + UMAP) used ABL1 transferase (UniProt: P00519) exclusively, with 3,325 ABL1 actives and 1,293,529 ZINC decoys (5 replicates).

For each experiment, we computed enrichment metrics (EF@1% and ROC-AUC) across multiple replicates and performed independent *t*-tests comparing Phase 5 (no UMAP) against Phase 1/4 baselines (with UMAP). Significance thresholds are set to *p<0.05, **p<0.01, ***p<0.001.

## Results

Results are reported in the same order as described in the previous section. It is important to understand what values the metrics would assume in the worst case, where the screening model would produce a uniform-randomly distributed ranking (see Table 1). In that case, the expected value of the first term in Eq. 3 is exactly 1.0. For Phases 1, 2, 3 and 5, the same expected BEDROC metric will result since the same number of actives (n=5,505) and total molecules (N=1,718,819) were used.

**Table 1:** Expected metric values for a random ranking based on set sizes used in Phases 1–3, 5

Metric	Expected Value
EF@1%	1.0000
BEDROC (α=20)	0.0516
BEDROC (α=160)	0.0080
ROC-AUC	0.5000
PR-AUC = $R_a$	0.0032

### Phase 1: hyperparameter optimization

Performance variation was determined through systematic evaluation of 515 hyperparameter configurations. This evaluation comprehensively covered two dimensionality reduction methods (PCA, UMAP) and two molecular representations (Mordred descriptors, ECFP4 fingerprints). The extensive hyperparameter grids revealed substantial performance variation.

**Fig. 1:**
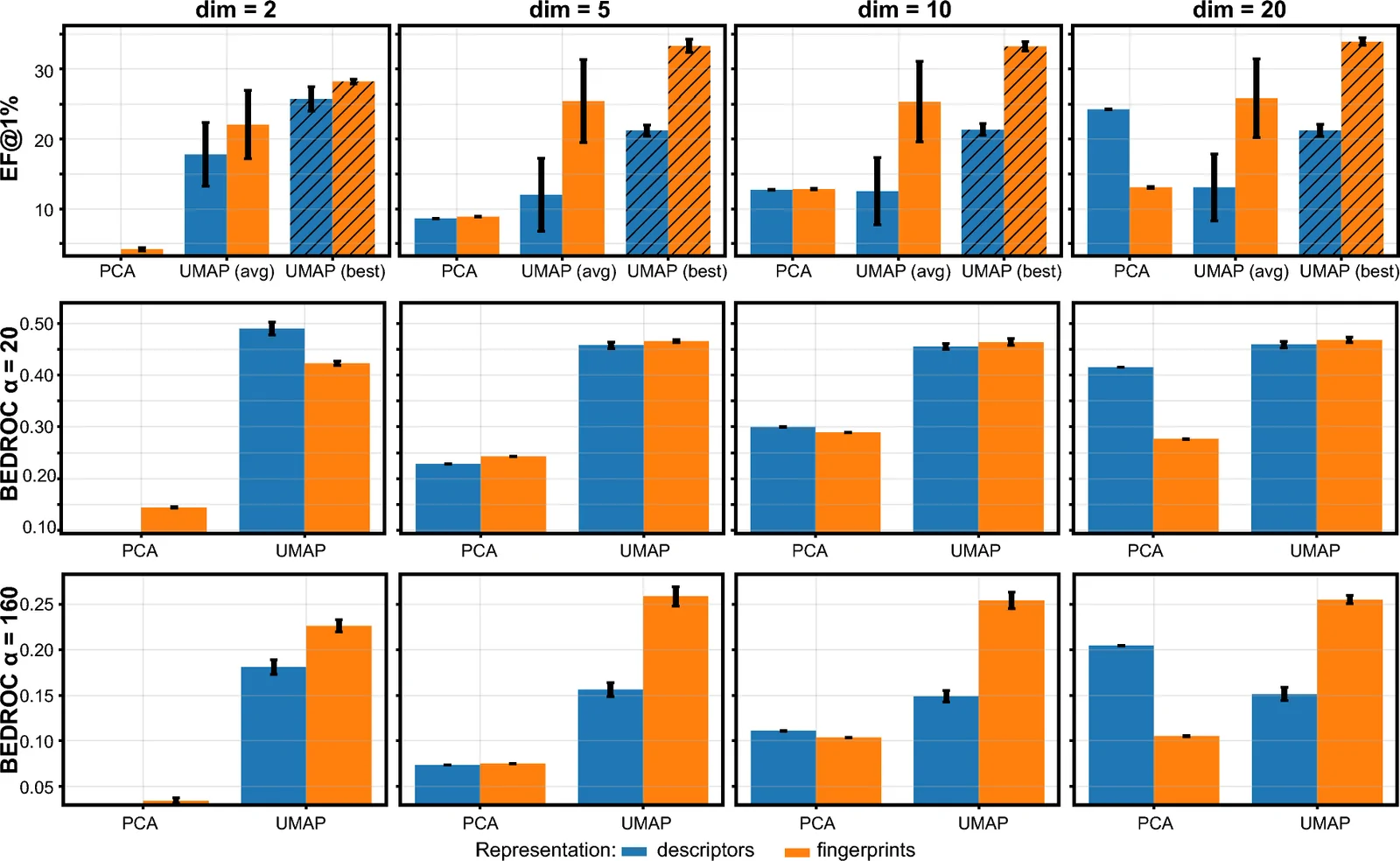
Phase 1—dimensionality-stratified EF@1% Comparison. EF@1%, BEDROC (α=20/160) across four embedding dimensionalities (2D, 5D, 10D, 20D) for PCA, UMAP-average (mean across all hyperparameter combinations), and UMAP-best (optimal hyperparameters). Blue bars: Mordred descriptors. Orange bars: ECFP4 fingerprints. Error bars represent standard deviation across replicates

#### Overall enrichment performance across dimensionalities

Examination of enrichment performance across embedding dimensionalities shows that ECFP4 fingerprints consistently outperform Mordred descriptors when evaluated on enrichment (Fig. 1). The optimal configuration for EF@1% was UMAP with ECFP4 fingerprints (20 dimensions, n_neighbors=10, min_dist=0.0, Jaccard metric), achieving a value of 33.94 ± 0.56. PCA baselines performed substantially worse. The best performance for descriptors was an EF@1% of 24.27 at 2D and for fingerprints an EF@1% of 13.08 at 20D), confirming that non-linear manifold learning is critical for enrichment.

Fingerprints’ structural encoding provides a consistent advantage across all dimensionalities for general retrieval. However, as detailed in the subsequent section, this trend starts to invert when specifically targeting high-potency compounds.

#### Potency-stratified analysis

An important shift in performance emerges when evaluating enrichment stratified by compound potency. While fingerprints dominate global metrics, Mordred descriptors coupled with UMAP achieve better enrichment for high-potency actives (affinity < 100 nM), at least relative to the mean enrichment over the entire set.

**Fig. 2:**
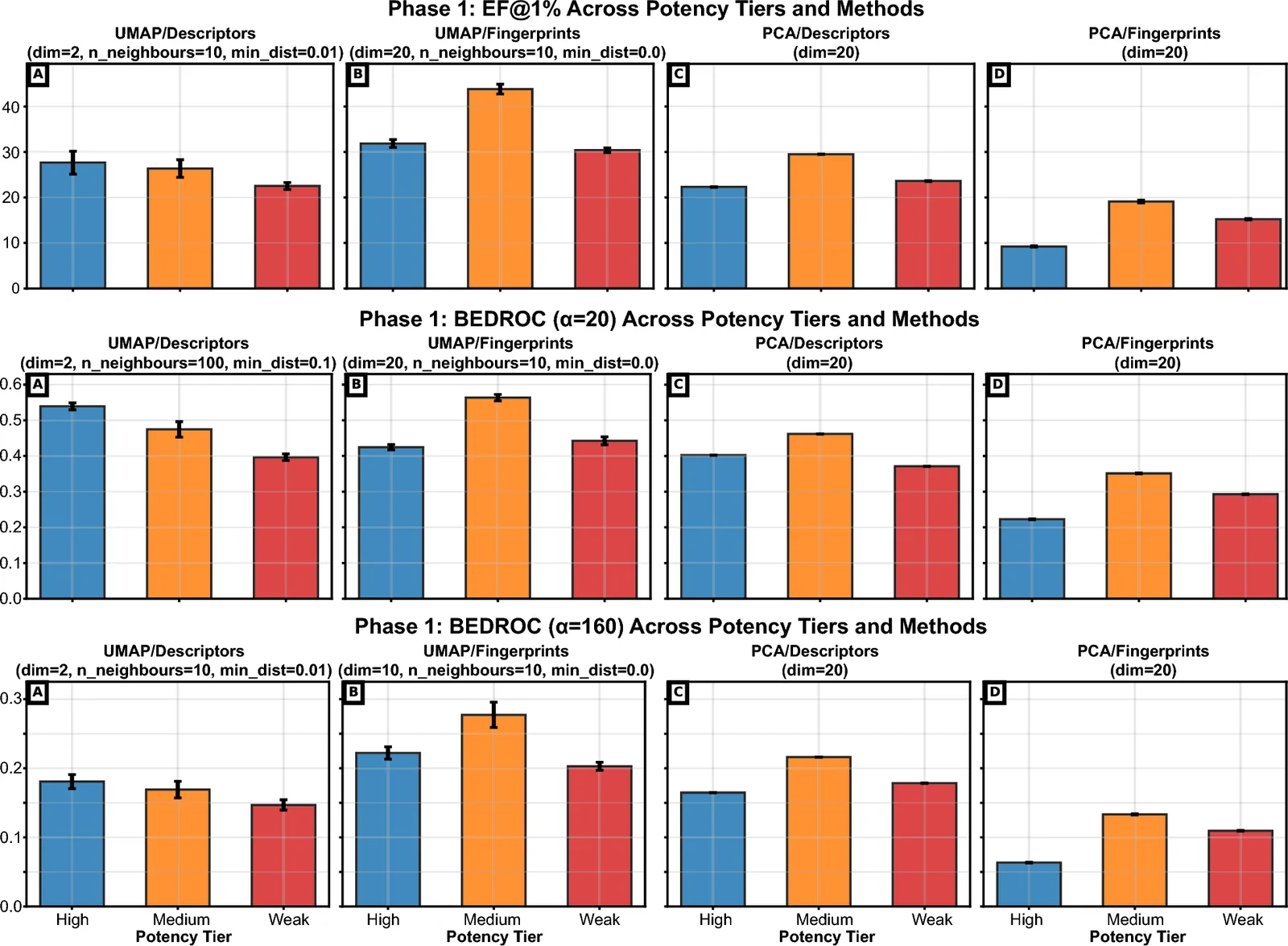
Phase 1—potency-stratified enrichment. EF@1%, BEDROC (α=20/160) for best-performing configurations across four method combinations, stratified by active potency tier (High: affinity < 100 nM, Medium: 100–1000 nM, Weak: 1000–10,000 nM). Error bars represent standard deviation across replicates

As shown in Fig. 2, the scores for Mordred descriptors on high-potency targets exceed those of medium and weak potency tiers. Conversely, ECFP4 fingerprints show better performance for the medium potency tier. This suggests that while fingerprints effectively retrieve topologically similar scaffolds, the physicochemical properties encoded by Mordred descriptors are better suited for clustering the tightest binders in the embedding space. However, fingerprints still seem to outperform descriptor-based UMAP similarity spaces.

#### Optimal hyperparameter landscape

**Fig. 3:**
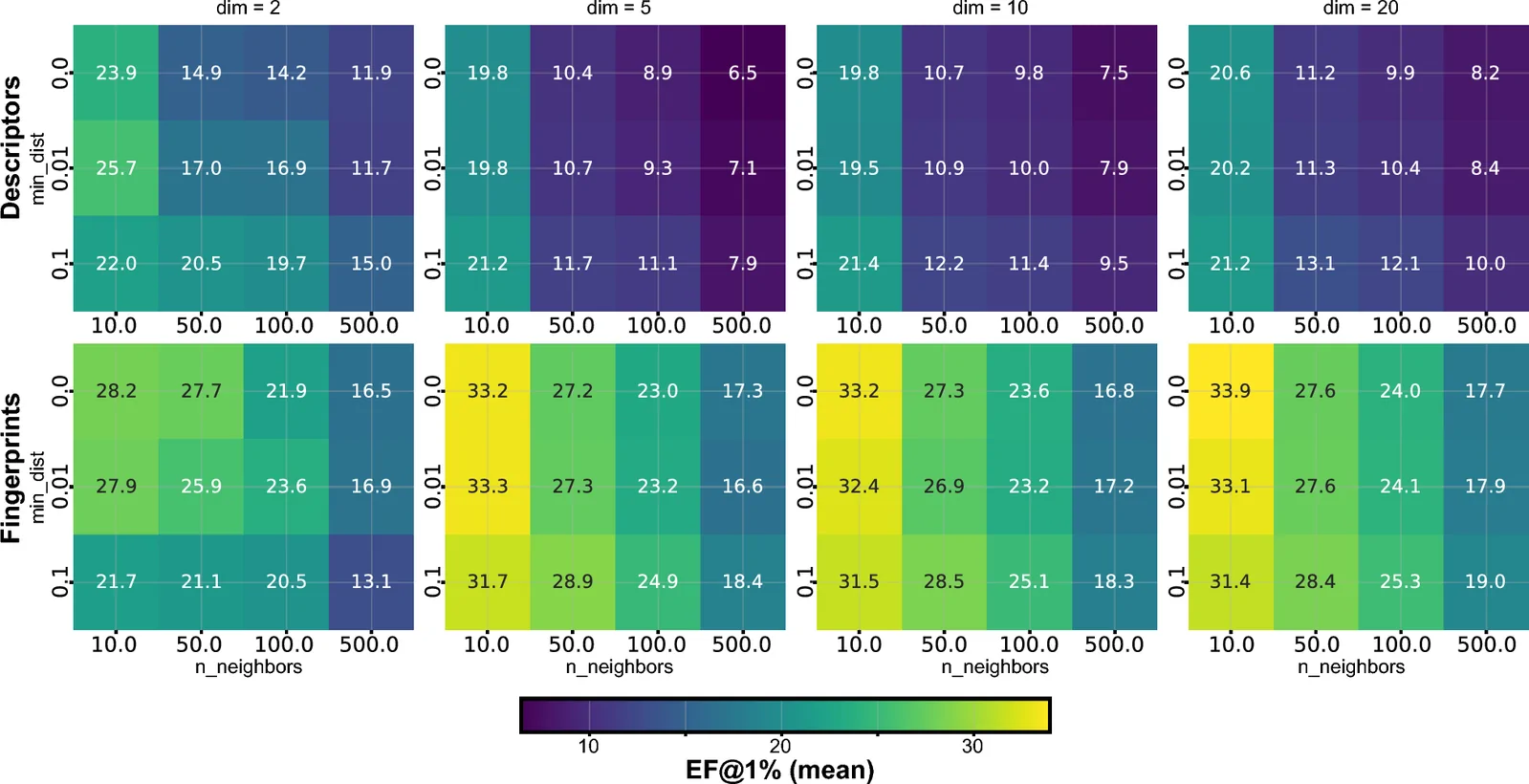
Phase 1—optimal hyperparameters and sensitivity. EF@1% values for all UMAP hyperparameter configurations. Low values for the number of neighbors (n neighbors=10–20) consistently performed best

Analysis of the 515-run grid (Fig. 3) revealed clear topological preferences for the embedding. Low values for the number of neighbors (n_neighbors=10–20) consistently performed best, indicating that preserving local neighborhood structures is more critical for 1-NN retrieval than preserving the global manifold geometry. Furthermore, tight clustering parameters (min_dist=0.0–0.01) outperformed dispersed embeddings (min_dist=0.1), suggesting that dense packing of the RS aids in creating discriminative decision boundaries for the 1-NN scorer.

### Phase 2: affinity cutoff sensitivity reveals optimal scoring against high-potency reference set

Using the optimal embeddings from Phase 1, we investigated the impact of RS quality on retrieval performance. We rescored held-out actives against subsets of the RS, filtered by varying affinity thresholds (100–100,000 nM).

**Fig. 4:**
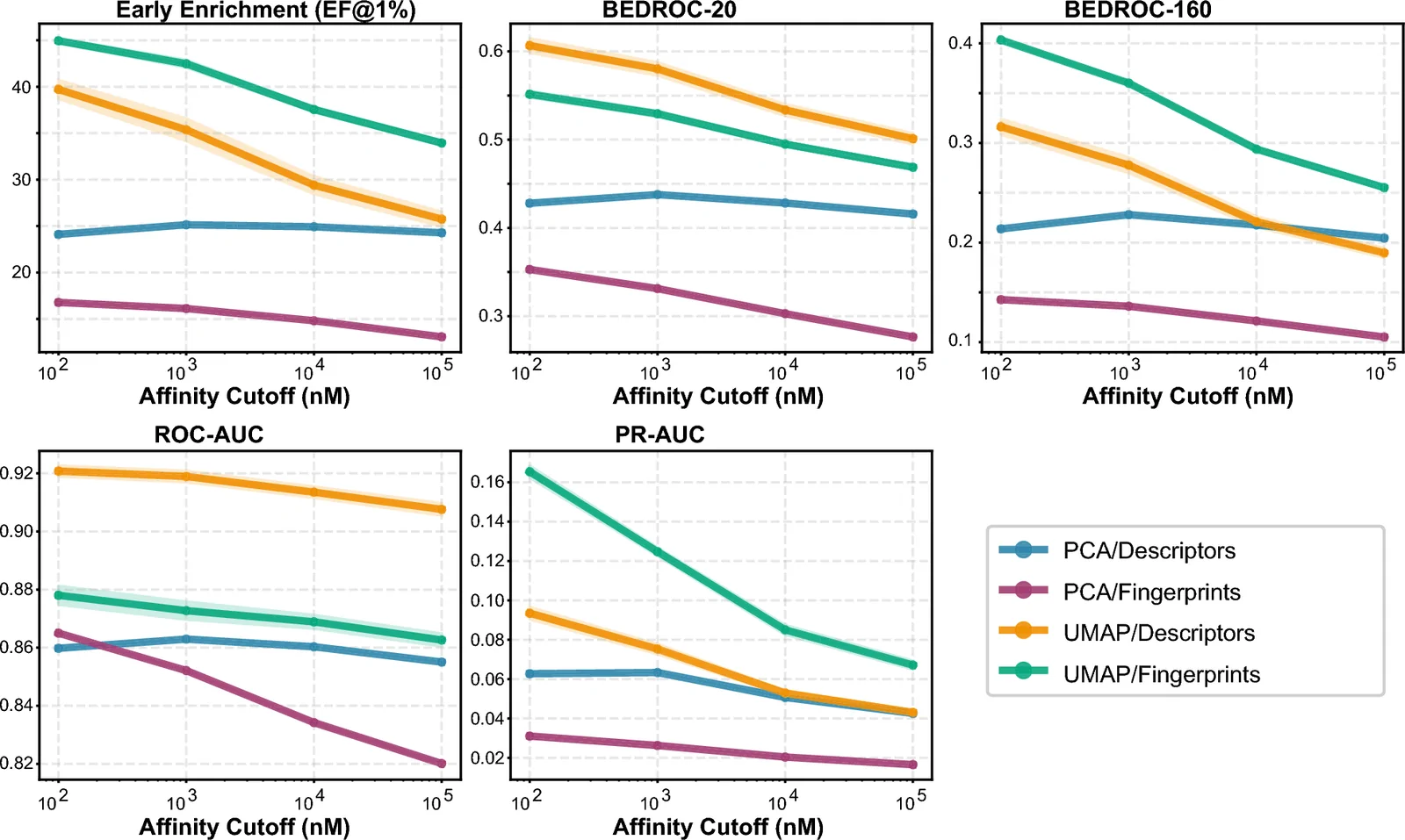
Phase 2—Affinity cutoff sensitivity for all metrics and methods, shaded area shows 95% confidence interval

**Fig. 5:**
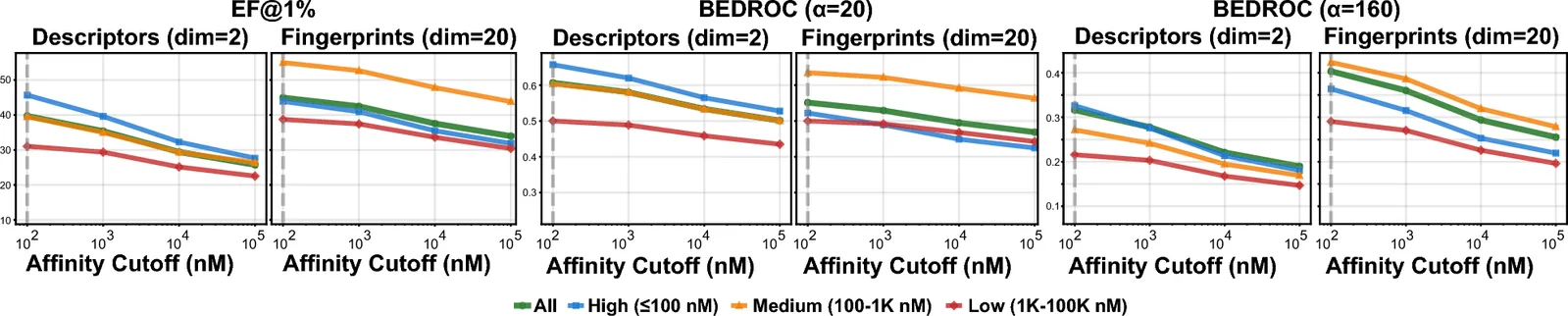
Phase 2—Affinity cutoff sensitivity for stratified potency sets using UMAP

Figure 4 shows a strong negative correlation between the inclusion of weak binders (affinity >1 μM) into RS and enrichment performance. Including weak binders creates “noise” in the embedding space, allowing decoys to score well by appearing similar to weak, non-specific binders. The same effect is also clearly seen in Fig. 5, where the performance for the different potency tiers of the target compounds is plotted. While medicinal chemistry programs routinely optimize weak initial hits when no alternatives exist, virtual screening benchmarks and early-hit workflows place a premium on prioritizing strong binders (blue line) over weak binders (red line). In benchmark evaluations, distinguishing potent from weak binders of the same target is formally defined and evaluated as “ranking power” [53]. From a practical screening standpoint, experimentally validating weak, micromolar-range hits requires elevated assay concentrations where non-specific artifacts such as colloidal aggregation are prevalent [51]. Conversely, computational prioritization of high-affinity matter seeks to directly identify cleaner, more verifiable starting points [34]. Descriptor-based UMAP similarity spaces outperform fingerprint-based similarity spaces in terms of EF@1% and BEDROC (α=20) scores but fingerprints still outperform descriptors for the stricter BEDROC (α=160) score.

One can also note that the choice of metric leads to different choices for UMAP, either for descriptors or fingerprints. Filtering the RS to include only high-potency compounds (< 100 nM) resulted in the highest enrichment across all metrics for the UMAP methods. Consequently, we adopted the 100 nM affinity cutoff as the default for subsequent experiments to maximize the signal-to-noise ratio in the reference manifold. PCA on descriptors is influenced the least by changing the filtering of RS and PCA on fingerprints is generally the worst performer. We therefore choose to ignore PCA in the rest of the experiments.

### Phase 3: reference set size ablation reveals robustness and critical point

**Fig. 6:**
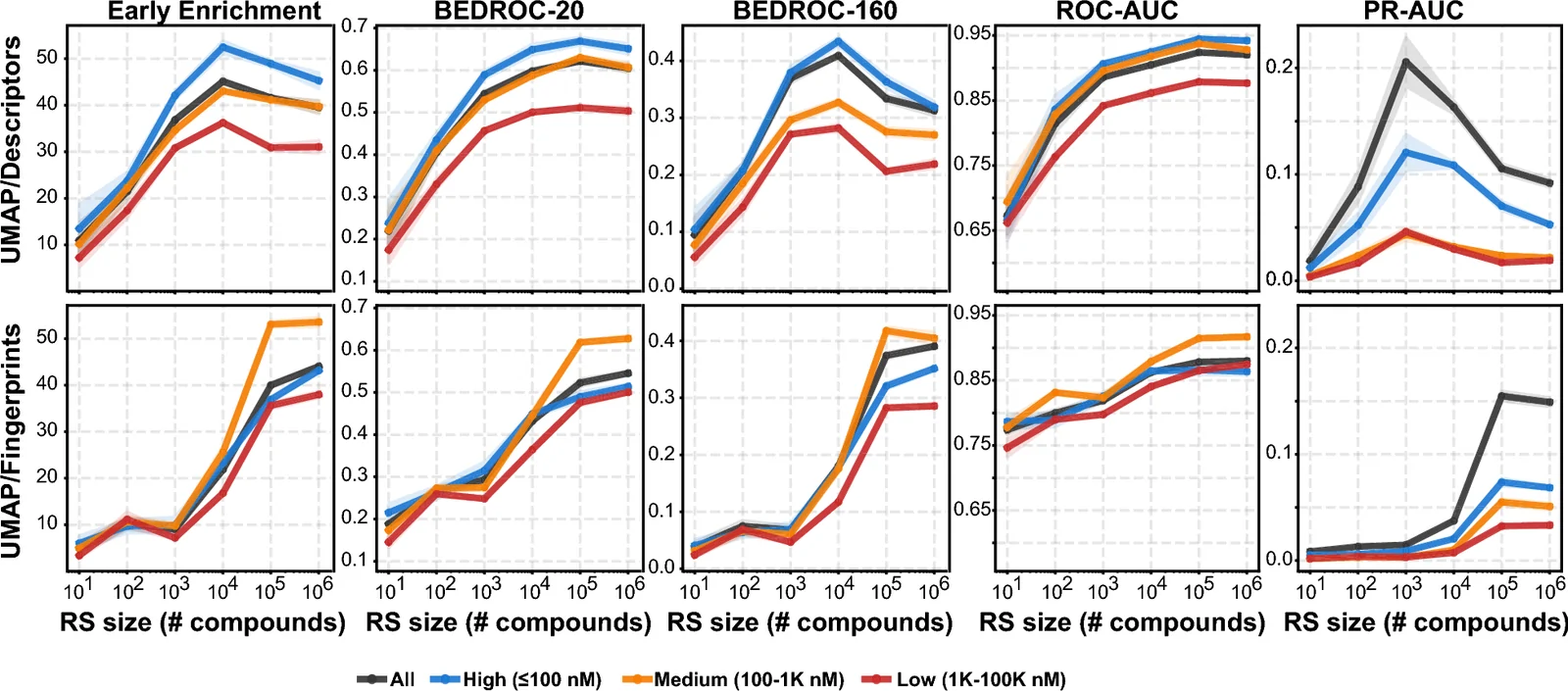
Phase 3—Sensitivity to RS size for all potency tiers, all metrics and methods. Metric values are plotted on y-axes

To test the limits of the “low-data” assumption, we systematically subsampled the RS from 10 to 100,000 (about 50,000 fewer than the original data set) compounds and retrained the models. Figure 6 illustrates the relationship between RS size and enrichment metrics, revealing a striking non-monotonic trajectory. At all RS sizes, Mordred descriptors outperformed ECFP4 fingerprints for high-potency retrieval, reinforcing the conclusion that rich physicochemical descriptor spaces better capture affinity-relevant features than structural fingerprints. These trends are consistent across all metrics employed.

*Robustness in Low-Data Regimes:* MolFuSE demonstrates remarkable efficiency with small datasets. Using UMAP on the descriptors of a small RS of just 1,000 compounds achieves an EF@1% of 41.77 ± 1.38. This strengthens the method’s applicability to orphan targets where limited bioactivity data is available.

However, contrary to the assumption that “more data is better”, when applying UMAP on descriptors, performance peaks at approximately 10,000 compounds (EF@1% 53.62 ± 1.70) and significantly degrades as the set size increases to 100,000+ compounds. This degradation (Fig. 6, right columns) suggests a descriptor space dilution effect. As the RS grows to encompass the full range of transferase inhibitor space, the UMAP embedding must accommodate increasingly diverse molecular structures. This forces the manifold to change in ways that disperse the high-potency clusters, making the 1-NN distance less discriminative. This finding highlights a critical trade-off between chemical space coverage and embedding specificity. Consequently, curating RS to exclude very weak binders (e.g., affinity > 10 μM) may improve performance, even if it reduces the total compound count.

### Phase 4: cross-target generalization

**Fig. 7:**
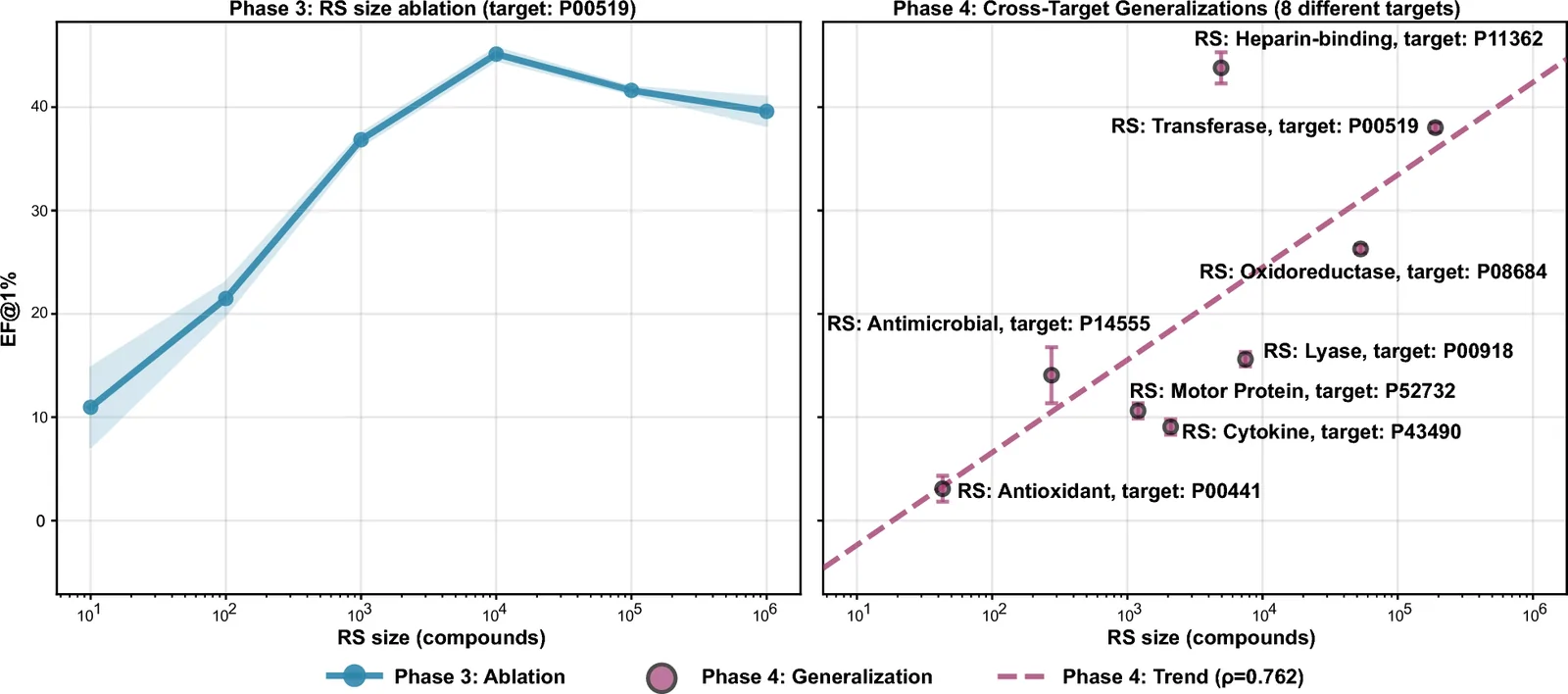
When using hyperparameters from Phase 3 (left), their generalization performance (EF@1%) is tested in Phase 4 (right) on other target proteins with other RS from other molecular functions

To ensure that the results are not artifacts of the ABL1 target, we applied the optimal configuration (i.e., UMAP 2D, Mordred, n_neighbors=10, min_dist=0.01, 100 nM cutoff) to eight additional functional classes with varying natural RS sizes (43,000 to 425,000 compounds). For each target protein’s data set, we trained a UMAP model. Figure 7 summarizes the cross-target performance. Phase 3 overestimated the generalization capability on smaller RS; however, the linear trend of the correlation between RS size and enrichment is clearly visible. Furthermore, the models were retrained without rehyperparameterizing UMAP for the other targets since this is a very computationally expensive operation.

Two additional notable observations include:

1. Consistency: The trend line (dashed red) indicates a positive correlation between available data and performance, although the gains plateau, mirroring the saturation effects seen in Phase 3.
2. Metric Stability: The method maintains high early enrichment (EF@1%) across diverse transferase sub-families, validating the generalizability of the manifold learning approach.

These results confirm that the parameters optimized on ABL1 transfer effectively to related protein targets, supporting the utility of MolFuSE as a algorithmic tool for protein functional class-wide screening.

### Phase 5: Ablation and Decoy Bias study

#### Database bias negative control

Table 2 shows that non-transferase actives scored against transferase-trained models achieved EF@1% = 13.5 ± 11.2 and ROC-AUC = 0.655 ± 0.107 across seven target families. This substantially exceeds random performance (EF@1% = 1.0, Table 1), confirming the presence of database bias: the model preferentially ranks ChEMBL compounds over ZINC decoys independent of target-specific biological relevance.

**Table 2:** Validation and baseline experiments versus phase 1/4 baselines

Method	EF@1%	ROC-AUC	p-value
*Database bias control*			
Non-kinase actives versus kinase model	13.5 ± 11.2	0.655 ± 0.107	N/A
*Descriptor comparison (raw descriptors)*			
Phase 5 (raw descriptors, NO UMAP)	7.8 ± 9.1	0.504 ± 0.121	–
Phase 4 (descriptors+UMAP)	20.7 ± 14.7	0.729 ± 0.130	< 0.001***
*Fingerprint comparison (tanimoto)*			
Phase 5 (raw ECFP4, NO UMAP)	30.1 ± 0.0	0.961 ± 0.000	–
Phase 1 (ECFP4+UMAP)	33.9 ± 0.5	0.863 ± 0.004	< 0.001***

This bias likely arises from two sources: (1) ChEMBL compounds are drug-like compounds with measured bioactivity, exhibiting restricted physicochemical properties (e.g., Lipinski’s rule of five), while ZINC contains broader chemical diversity that includes non-drug-like structures; (2) RS itself is derived from ChEMBL, creating implicit training set homogeneity. Future work should explore alternative decoy sets (e.g., property-matched DUD-E decoys) to isolate target-specific signals more cleanly, though such matched sets introduce their own biases.

However, it is important to note that this bias effect (EF@1% = 13.5) is approximately 2.5-fold *lower* than target-specific performance (EF@1% = 30–34 for matched transferase targets). This quantitative difference demonstrates that the ChEMBL and ZINC databases are only *partially* separable, not fully trivially distinguishable. Target-specific biological signals clearly dominate MolFuSE’s predictions. The model is not merely learning to distinguish “ChEMBL-like” from “ZINC-like” compounds, but instead capturing genuine protein-ligand interaction patterns superimposed on a non-negligible background of database bias.

#### Raw descriptor ablation

Using the raw 1,611-dimensional descriptors without UMAP achieved an EF@1% of 7.8 ± 9.1 and ROC-AUC of 0.504 ± 0.121 across the 7 target families (Table 2). In contrast, the descriptors + UMAP achieved an average EF@1% = 20.7 ± 14.7 and ROC-AUC = 0.729 ± 0.130 over the same targets and descriptors (p<0.001, 166% relative improvement). These results reflect average behavior across all target families, capturing UMAP ’s generalizable benefit rather than target-specific tuning. This improved performance demonstrates that UMAP is not merely a computational convenience but is necessary for effective descriptor-based screening.

The poor performance of using only high-dimensional descriptors can be attributed to the curse of dimensionality: in 1,611-dimensional space, the Euclidean distance metrics become uninformative as all pairwise distances converge [5]. The nearest-neighbor search in such spaces approaches random selection because the ratio of nearest-to-farthest neighbor distances tends toward 1.0. UMAP addresses this by learning a low-dimensional manifold that preserves local neighborhood structures, effectively denoising the descriptor space and recovering biologically meaningful similarity relationships. Without this manifold learning step, the high-dimensional descriptor space is notably less useful in ligand-based screening.

#### Fingerprint ablation

Raw ECFP4 fingerprints with Tanimoto similarity (i.e., without UMAP) achieved EF@1% = 30.1 ± 0.0 and ROC-AUC = 0.961 ± 0.000 (Table 2). This represents strong performance and establishes a high baseline for comparison. Completion of Phase 1 (ECFP4 + UMAP) achieved an EF@1% = 33.9 ± 0.5 (p<0.001), an 11% improvement, and a ROC-AUC = 0.863 ± 0.004. Comparison of these metric values shows an unusual pattern: raw ECFP4 has higher global ranking quality ($\Delta \text {ROC-AUC} = +0.1$; +10%). In contrast, ECFP4 + UMAP has superior early enrichment ($\Delta EF@1\% =+3.8$; +11%). This indicates UMAP prioritizes top-ranked predictions—the most critical region for practical virtual screening where experimental validation costs dominate. The trade-off is acceptable: a 10% reduction in global AUC is offset by an 11% gain in the actionable top 1% of predictions.

### MolFuSE explorer

**Fig. 8:**
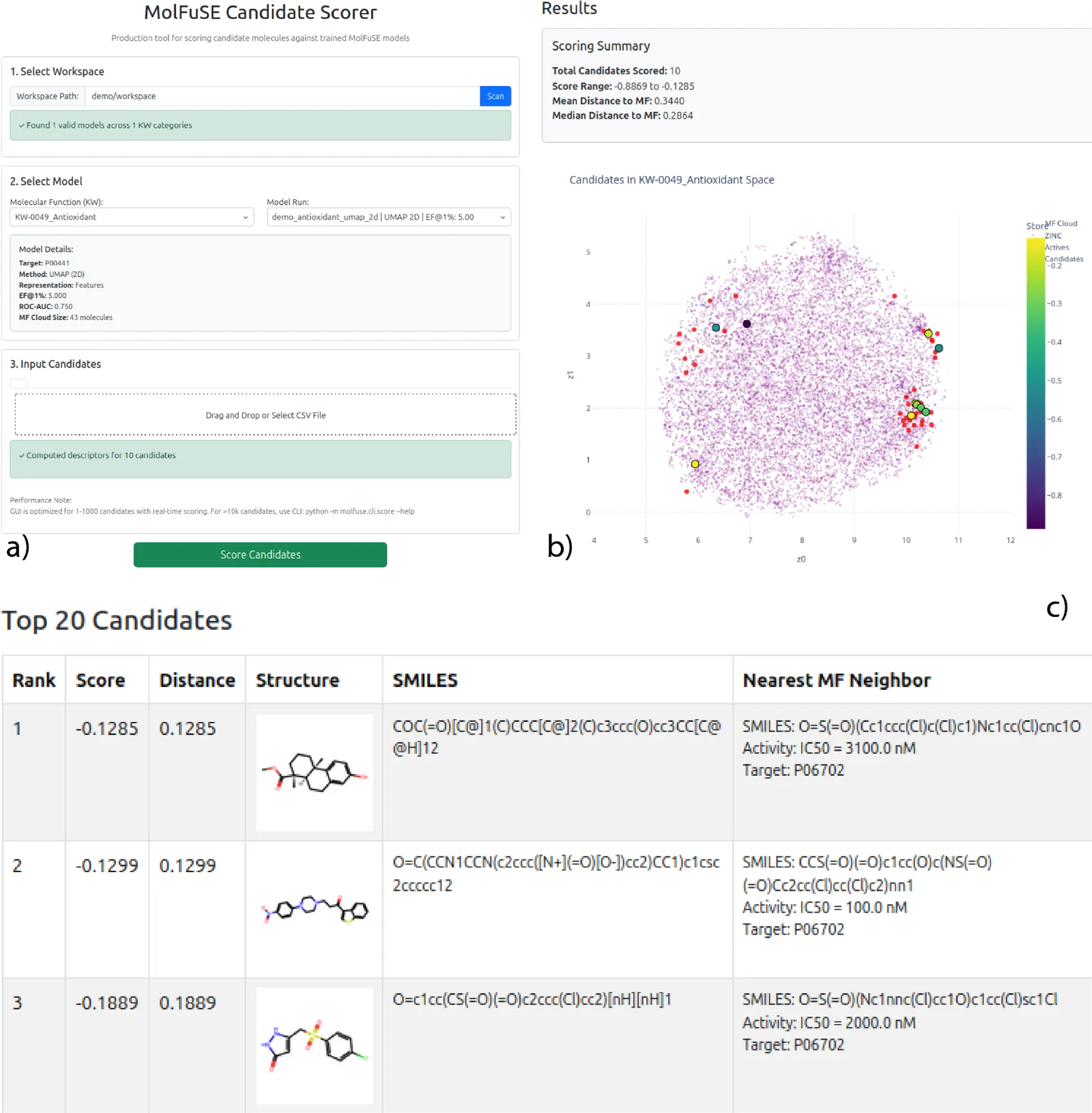
The MolFuSE Explorer. **a** User selects workspace, model and input candidates. **b** The resulting similarity space is calculated by the backend. **c** Candidates are scored, and top candidates are revealed

Akin to tools like the SwissSimilarity web tool [62], which predicts the targets of bioactive compounds based on a combination of 2D and 3D similarity measures and linear projections with known ligands, we introduce a web application, shown in Fig. 8, designed for interactive MolFuSE navigation and analysis of the RS-guided similarity spaces. This goes beyond applications such as VSFlow [22] that do not involve dimensionality reduction techniques.

The primary interface is an interactive 2D scatter plot that visualizes the selected space. Points are color-coded to distinguish between known binding ligands for the functional class, ZINC decoys, and user-uploaded compounds. Users can dynamically filter the displayed data based on activity thresholds and other properties. A key capability is the projection of user-uploaded custom compounds into the current RS-guided space using a trained UMAP model. This allows users to assess their novel compounds in the context of known RS-relevant actives and the broader purchasable chemical space. To aid candidate identification, a 1-Nearest Neighbors (1-NN) search can be performed within the 2D space to find the most similar ZINC or custom compounds to the aggregated set of ChEMBL actives.

## Discussion

This study demonstrates that in low-data virtual screening regimes, high-performing prediction models can be developed using manifolds learned from homologous protein data. Our findings regarding potency stratification, data saturation, and embedding dimensionality suggest that MolFuSE operates more robustly through a similarity-based scoring mechanism governed by physicochemical compatibility (using Mordred descriptors) rather than pure substructural analogy ECFP4 fingerprints, although the latter encoding strategy should always be considered, especially with larger RS sizes.

### Physicochemical descriptors drive high-potency early enrichment

A central finding in the hyperparameter and representation analysis (Phase 1) is the performance divergence between topological fingerprints (ECFP4) and physicochemical descriptors (Mordred) when evaluated against high-potency targets. While fingerprints generally offer robust global retrieval—evidenced by high ROC-AUC scores in baseline comparisons—descriptor-based UMAP embeddings demonstrated a specific advantage in concentrating sub-100 nM binders at the very top of the active rankings. This advantage, reflected in the higher high-potency discrimination of descriptor-based UMAP embeddings at optimized RS sizes, suggests that tight binding is driven by specific physicochemical property profiles that are explicitly encoded by Mordred descriptors, whereas fingerprints excel at broader structural retrieval. While fingerprints excel at identifying structural analogs (scaffold hopping), descriptor-based manifold learning appears to filter the potency better, resulting in clustering compounds with the requisite physical properties for tight binding regardless of their scaffold similarity, especially when RS size is small. This distinction supports the use of descriptor-based embeddings specifically for the early recognition problem, where limited experimental data hinders the prediction of top-ranked candidates.

### Descriptor space dilution and the limits of data scaling

Contrary to the prevailing big data paradigm in deep learning, where performance typically scales with dataset size, our reference set (RS) ablation studies (Phase 3) identified a critical performance ceiling. The observation that enrichment peaks at an RS size of approximately 10,000 compounds and significantly degrades as the set expands to 100,000+ provides a nuanced view of the “Similar Property Principle” [52] in low-dimensional embeddings. We attribute this degradation to descriptor space dilution. As the RS grows to encompass the historical diversity of a functional class’s active compounds, it inevitably incorporates a wider array of distinct molecular structures and weaker binders. In the context of a fixed-dimensional UMAP embedding, preserving the global structure of this heterogeneous data forces the manifold to expand, dispersing the dense local clusters of high-potency compounds required for effective 1-NN retrieval. This is further supported by Phase 2 results, where excluding weak binders (≥100 nm) from the scoring set improved performance. Consequently, for embedding-based similarity search, the quality and physicochemical specificity of the reference data appear more critical than volume. This finding is particularly relevant for “low-data” targets, as it implies that massive historical datasets are not required if a smaller, curated set of highly specific ligands is available.

Dimensionality reduction: necessity and trade-offs The ablation study clarifies the role of UMAP not merely as a visualization tool, but as a necessary pre-processing step for physicochemical screening. The failure of raw high-dimensional descriptors (EF@1% ≈7.8) compared to their UMAP-projected counterparts (EF@1% ≈20.7) confirms that the “curse of dimensionality” renders Euclidean metrics ineffective in the raw 1,611-dimensional space. UMAP mitigates this by learning a manifold that preserves local neighborhood structures, effectively denoising the descriptor space. However, this gain in early enrichment comes at the cost of global ranking quality. While the ECFP4-based MolFuSE workflow improves early enrichment EF@1% by 11% over the raw Tanimoto baseline, the descriptor-based approach provides more actionable prioritization of high-potency hits at the cost of a lower global ROC-AUC. This trade-off is consistent with UMAP’s algorithmic focus on local versus global structure preservation. For practical virtual screening, where only the top 0.1% to 1% of a library is typically screened, optimizing for EF@1% and BEDROC is operationally more valuable than maintaining a correct ranking order among non-active decoys.

### Database bias and generalization

Finally, the detection of a moderate baseline preference for ChEMBL compounds over ZINC decoys underscores the challenge of database bias in retrospective validation. While the target-specific biological signal was found to be approximately 2.5-fold stronger than this background bias, absolute performance metrics must be interpreted with this inflation in mind. Despite this, the consistent performance across eight diverse protein targets (Phase 4) demonstrates that the method generalizes beyond the optimization target (ABL1), with performance dropping with the size of RS. It should be noted that the cross-target validation was performed without rehyperparameterizing UMAP for each specific functional class; thus, the reported results likely represent a conservative lower bound of the method’s potential performance when tuned for specific target families.

The Phase 5 controls establish three key findings:

1. Partial database bias exists but does not dominate. MolFuSE exhibits moderate ChEMBL-over-ZINC enrichment for irrelevant targets (EF@1% = 13.5), confirming database provenance introduces bias. However, this effect is 2.5-fold weaker than target-specific performance (EF@1% = 30–34), demonstrating that biological signals dominate over database artifacts. Absolute performance metrics should be interpreted with this bias acknowledged.
2. UMAP is essential for descriptor-based screening. Compared to the 1,611-dimensional raw descriptors (EF@1% = 7.8), UMAP embeddings increase performance by 166% (EF@1% = 20.7, p<0.001). The curse of dimensionality renders high-dimensional Euclidean spaces ineffective for nearest-neighbor search; manifold learning is necessary, not optional. Notably, Phase 4 UMAP results represent average cross-target performance, suggesting this benefit generalizes across protein families.
3. MolFuSE modestly improves upon industry-standard Tanimoto. Raw ECFP4 fingerprints with Tanimoto similarity provide a strong baseline (EF@1% = 30.1), which UMAP integration improves by 12.6% (EF@1% = 33.9, p<0.001). The gain is concentrated in early enrichment (top 1%), the most critical region for experimental validation. While UMAP introduces a trade-off by reducing global ROC-AUC (0.863 vs. 0.961), it notably improves actionable, top-ranked predictions, representing a good compromise for practical screening applications.

These results validate MolFuSE’s core hypothesis, that manifold learning enhances ligand-based virtual screening, while honestly characterizing limitations and establishing that simpler baselines (Tanimoto) remain competitive.

## Conclusions

Herein, we introduced MolFuSE, a projection-based virtual screening framework (i.e., for ligand-repurposing) that leverages a reference set (RS) of ligands that bind proteins with the same molecular function as the target protein. By capitalizing on UMAP’s dimensionality reduction, this method constructs a lower-dimensional embedding space that significantly enhances early recognition performance, achieving good enrichment of high-potency compounds in low-data regimes. Through systematic evaluation across four experimental phases, we demonstrate that this model has:

1. High-potency discrimination: UMAP embeddings of Mordred 2D molecular descriptors’ explicit encoding of binding-relevant properties (LogP, hydrogen bonding, electronic distribution) substantially outperforms topology-focused ECFP4 fingerprint-based methods in the early recognition (EF@1%) of tight binders, although topological fingerprints maintain better global ranking quality (ROC-AUC).
2. RS size vs. performance: Effective screening is achievable with RS as small as 1,000 compounds, enabling application to orphan targets and understudied protein families. Performance peaks at 10,000 compounds and degrades at 100,000+, suggesting that feature space dilution from structurally diverse weak binders impairs retrieval—challenging the assumption that larger RS are universally better. Instead, MolFuSE refines the ’similar property principle’ [52] by demonstrating that for high-potency retrieval, the quality and physicochemical specificity of RS are more critical than its size.

MolFuSE addresses a challenging problem in early-stage drug discovery: effective virtual screening for targets with limited SAR data. By transferring knowledge from related functional classes via RS, MolFuSE achieves good enrichment performance without direct knowledge of known binders. The method’s interpretability—via exact 1-NN scoring and traceable similarity paths—further enhances its utility for medicinal chemistry hit triaging.

### Limitations and future directions

While MolFuSE demonstrates robust performance, several avenues for refinement remain. First, UMAP hyperparameters were optimized to a single protein target and then held constant across all targets to ensure a standardized benchmark. Consequently, target-specific rehyperparameterization could yield improved results. While we also identified a bias in the decoy dataset, its impact on MolFuSE’s performance was minimal.

A primary limitation is the reliance on Mordred’s 2D descriptors:

- Shape and Stereochemistry: The current model does not explicitly encode 3D volume/curvature, shape moments, or chiral centers (i.e., enantiomers may receive identical embeddings).
- Conformational Flexibility: Conformer preferences and flexibility are currently unrepresented.

All results in this study are retrospective. While high enrichment of ChEMBL-sourced actives is valuable, the ultimate utility of MolFuSE must be confirmed through prospective experimental validation. Future efforts will focus on the testing of compounds to confirm that top-tier enrichment translates into viable experimental hit rates (>10%) and successful lead optimization campaigns. MolFuSE could be extended to look at off-target effects and toxicity by finding similar compounds with a shared scaffold but differing substitution patterns. It could also be used to suggest molecules that balance substructure similarity and differing extended/branching scaffolds. This next step would be a multi-objective LBVS strategy. In conclusion, by leveraging non-linear manifold learning, MolFuSE offers a beneficial path to accelerate hit discovery in data-constrained virtual screening workflows, particularly for under-explored therapeutic targets.

**Supplementary information** Please find an extended version of the related work and a full listing of the molecular descriptors in the SI.

## Supplementary Information

Supplementary file 1

## Data Availability

The complete computational pipeline (configuration generators, HPC batch scripts, post-processing analysis tools) is available at: https://github.com/m3-center/molfuse.git. The dataset and an example of a good UMAP model can be found at https://zenodo.org/records/20325360

## Footnotes

1 See https://ftp.uniprot.org/pub/databases/uniprot/current_release/knowledgebase/complete/docs/keywlist.txt for a full list of molecular function keywords.
