# Supplementary material for: Molecular function-guided unsupervised similarity-based enrichment for low-data virtual screening
Source: J Cheminform. 2026 Nov 6;18:156. doi: 10.1186/s13321-026-01326-y (PMC13750033; doi:10.1186/s13321-026-01326-y)
Supplement: Supplementary file 1 [file 13321_2026_1326_MOESM1_ESM.pdf]

Supplementary Material for: “Molecular Function-guided Unsupervised Similarity-based Enrichment for Low-Data Virtual Screening”

Alexander Hagg[1,2*], Dirk Reith[1,2,3], Matthias Preller[2,4,5], Karl N. Kirschner[2,6]

[1*]Department of Engineering and Communication, University of Applied Sciences Bonn-Rhein-Sieg, Grantham-Allee 20, Sankt Augustin, 53757, Germany.
[2*]Institute of Technology, Resource and Energy-efficient Engineering, University of Applied Sciences Bonn-Rhein-Sieg, Grantham-Allee 20, Sankt Augustin, 53757, Germany.
[3]Fraunhofer Institute for Algorithms and Scientific Computing, Schloss Birlinghoven, Sankt Augustin, 53754, Germany.
[4]Department of Natural Sciences, University of Applied Sciences Bonn-Rhein-Sieg, Von-Liebig-Straße 20, Rheinbach, 53359, Germany.
[5]Institute for Functional Gene Analytics, University of Applied Sciences Bonn-Rhein-Sieg, Von-Liebig-Straße 20, Rheinbach, 53359, Germany.
[6*]Department of Computer Science, University of Applied Sciences Bonn-Rhein-Sieg, Grantham-Allee 20, Sankt Augustin, 53757, Germany.

*Corresponding author(s). E-mail(s): alexander.hagg@h-brs.de;
Contributing authors: dirk.reith@h-brs.de; matthias.preller@h-brs.de; karl.kirschner@h-brs.de;

1 Extended Related Work

This section provides extended context on virtual screening metrics, baseline screening performance, deep learning architectures, and dimensionality reduction methodologies as documented in recent literature.

1.1 Metrics and Benchmarks in Virtual Screening

Quantifying the ability of virtual screening methods to prioritize active compounds – known as enrichment – is fundamental to evaluating their utility in early-stage drug discovery. While the literature presents a diverse array of evaluation metrics and benchmark datasets McGaughey et al. (2007); Sciabola et al. (2022), which often complicate direct cross-study comparisons, robust metrics such as the enrichment factor at 1% (EF@1%) have become standard for assessing early retrieval success. Since the datasets used in previous studies differ from the molecular function-guided configuration employed here, the performance figures below provide a general context for indirect comparison rather than a direct benchmark.

A critical analysis of structure-based virtual screening (structure-based virtual screening (SBVS)) benchmarks highlight that established resources such as the Directory of Useful Decoys (DUD) and its enhanced version (DUD-E) Mysinger et al. (2012) exhibit severe analogue and decoy biases Rohrer and Baumann (2009). These systematic biases can inflate performance estimates by 2–5 times relative to more recent, unbiased benchmarks such as LIT-PCBA Tran-Nguyen et al. (2020).

1.2 Advanced Virtual Screening Scoring Functions and Ensembles

When experimental target data is known, specialized scoring functions have reported high enrichments on standardized benchmarks. For example, the Similarity of Interaction Energy VEctor Score (SIEVE-Score), which incorporates molecular docking within its workflow, achieved a median EF@1% of 44.9 (10%/90% percentiles [27.3, 60.0]) on a subset of DUD-E targets, substantially outperforming classical Glide docking Yasuo and Sekijima (2019). In comparison, the machine-learned RF-Score on the same subset yielded median EF@1% values of 18.7 (10%/90% percentiles [4.1, 43.0]) and 38.6 (10%/90% percentiles [13.8, 58.9]) depending on training configuration Yasuo and Sekijima (2019).

Hybrid screening approaches have also demonstrated elevated performance. A protocol combining ensemble docking with electrostatic comparisons (e-Sim) Cleves et al. (2019) achieved a median EF@1% of 46.1 across a 96-protein DUD-E subset Cleves and Jain (2020). Furthermore, the ensemble learning method ENS-VS – which integrates support vector machines (support vector machines (SVMs)), a decision tree, and a Fisher linear discriminant – achieved a median EF@1% of 57.0 (10%/90% percentiles [51.2, 63.9]) on DUD-E, compared to AutoDock Vina alone which reached 4.7 (10%/90% percentiles [3.3, 17.7]) Li et al. (2020). When evaluated on the DEKOIS dataset Vogel et al. (2011), ENS-VS yielded a median EF@1% of 29.5 (10%/90% percentiles [21.2, 37.9]), compared to Vina (2.4), Glide (9.5), SIEVE-Score (28.1), and RF-ScoreVS_v3_vina (16.6) Li et al. (2020).

1.3 Deep Learning Representation Models

Modern deep learning architectures focus on navigating high-dimensional molecular descriptor spaces or generating latent representations of these spaces. Recent Graph

Neural Networks (GNNs) combining learned topologies with expert-curated descriptors have been trained on large-scale bioactivity datasets containing between 60,000 and 350,000 compounds Liu et al. (2025).

Similarly, Transformer models adapted for chemistry learn fundamental structural rules through pre-training on millions of unlabeled chemical structures Mswahili and Jeong (2024); Banerjee et al. (2025); Umer et al. (2025). Models like ChemBERTa Chithrananda et al. (2020) are designed for fine-tuning on diverse downstream tasks. Another prominent transformer model, MolBERT Fabian et al. (2020), designed to produce generalized molecular representations, achieved a BEDROC score of 0.344 ± 0.062 across 69 unique protein datasets Fabian et al. (2020). While computationally powerful, these frameworks generally demand substantial labeled training data and high-performance computing resources, and their internal mechanisms remain highly complex to interpret compared to distance-based metric retrieval.

1.4 Dimensionality Reduction Paradigms in Cheminformatics

Chemical descriptor packages often generate highly multidimensional representations. Dimensionality reduction is critical to compress these spaces, extract latent chemical features, and permit visualization Nguyen and Holmes (2019).

Linear dimensionality reduction techniques like Principal Component Analysis (principal component analysis (PCA)) generate embeddings by projecting data along directions of maximal variance Oprea and Gottfries (2001). However, because PCA relies entirely on linear transformations, it frequently struggles to maintain local, non-linear neighborhood structures that dictate molecular similarity and biological activity Orlov et al. (2025); Lovrić et al. (2021).

Conversely, non-linear techniques like t-distributed Stochastic Neighbor Embedding (t-SNE) Van der Maaten and Hinton (2008) and Uniform Manifold Approximation and Projection (uniform manifold approximation and projection (UMAP)) McInnes et al. (2018) resolve some of these limitations but utilize different mathematical frameworks. t-SNE focuses heavily on preserving local distances, which often causes global spatial relationships and inter-cluster distances between distinct chemotypes to lose quantitative meaning Kobak and Berens (2019). In contrast, UMAP relies on Riemannian geometry and algebraic topology, allowing it to preserve local neighborhoods while maintaining global structure. This balance makes relative cluster positioning more physically interpretable. Furthermore, UMAP is computationally faster, scales more effectively to large datasets, and offers standard formulations to project novel, out-of-sample molecules into an existing embedding space without requiring model retraining Becht et al. (2019).

While manifold learning approaches are routinely utilized for visual representation Probst and Reymond (2020); Cihan Sorkun et al. (2022); Orlov et al. (2025) and clustering Hernández-Hernández and Ballester (2023); Guo et al. (2024, 2025), their application as a direct distance-based scoring metric for virtual screening retrieval remains a relatively underexplored paradigm compared to classical fingerprint comparisons or linear projections.

2 Molecular Descriptors

The 1,611 Mordred 2D molecular descriptors used in this work are organised into twelve categories in Tables 1–2 and 3–6.

The autocorrelation weight codes used throughout are: c = Gasteiger charge, dv = valence electrons, d = sigma electrons, s = intrinsic state, Z = atomic number, m = atomic mass, v = van-der-Waals volume, se = Sanderson electronegativity, pe = Pauling electronegativity, are = Allred–Rochow electronegativity, p = polarizability, i = first ionization potential.

Table 1 Constitutional Descriptors.

**Descriptor(s)**	**Description**
MW, AMW	Molecular weight and average molecular weight.
$nAtom$, $nHeavyAtom$, nH	Total atom count, heavy atom count, hydrogen count.
$nHetero$, nX	Heteroatom and halogen counts.
nB, nC, nN, nO, nS, nP	Per-element atom counts (B, C, N, O, S, P).
nF, nCl, nBr, nI	Halogen atom counts (F, Cl, Br, I).
$nAromAtom$, $nAromBond$	Aromatic atom and bond counts.
$nSpiro$, $nBridgehead$	Spiro and bridgehead atom counts.
$nAcid$, $nBase$	Number of acidic and basic functional groups.
$nHBAcc$, $nHBDon$	Hydrogen-bond acceptor and donor counts.
$nRot$, $RotRatio$	Rotatable bond count and ratio.
$nBonds$, $nBondsO$, $nBondsS$	Total bond count, single bonds, and bond-order sum.
$nBondsD$, $nBondsT$, $nBondsA$	Double, triple, and aromatic bond counts.
$nBondsM$, $nBondsKS$, $nBondsKD$	Multiple, Kekulé-single, and Kekulé-double bond counts.
$RNCG$, $RPCG$	Relative negative/positive charge (most charged atom fraction).
$FCSP3$, $HybRatio$	Fraction of sp^3 carbons; ratio of sp^3 atoms to heavy atoms.
$C1SP1$–$C4SP3$	Carbon counts by hybridization state and substitution (sp, sp^2, sp^3).
$fragCpx$, fMF	Fragment complexity and molecular framework fraction.
$apol$, $bpol$	Sum of atomic and bond polarizabilities.
$VMcGowan$, $Vabc$, $VAdjMat$	McGowan characteristic volume, ABC volume, adjacency-matrix volume.
$LabuteASA$	Labute approximate surface area.

Table 2 Graph-Theoretical and Physicochemical Descriptors.

Descriptor(s)	Description
ABC, $ABCGG$	Atom-Bond Connectivity index and Graovac–Ghorbani variant.
$BalabanJ$	Balaban's connectivity (J) index.
$WPath$, $WPol$	Wiener path index and Wiener polarity index.
$Zagreb1$, $Zagreb2$, $mZagreb1$, $mZagreb2$	First and second Zagreb indices and their modified variants.
$BertzCT$	Bertz complexity index.
$ECIndex$	Eccentric connectivity index.
$DetourIndex$	Detour index (longest-path sum).
$Diameter$, $Radius$	Topological diameter and radius.
$TopoShapeIndex$, $PetitjeanIndex$	Topological and Petitjean shape indices.
$SLogP$, SMR	Wildman–Crippen $\log P$ and molar refractivity estimates.
$FilterItLogS$	Filter-it predicted aqueous solubility ($\log S$).
$TopoPSA$, $TopoPSA(NO)$	Topological polar surface area (all heteroatoms; N/O only).

Table 3 Autocorrelation Descriptors. Weight codes $w \in \{c, dv, d, s, Z, m, v, se, pe, are, p, i\}$ (see text); lag k ranges as noted.

Category	Descriptor(s)	Description
**Autocorrelation**	$ATS_k w$	Moreau–Broto autocorrelation, lag k = 0–8; weights $w \in \{dv, d, s, Z, m, v, se, pe, are, p, i\}$.
	$AATS_k w$	Average Moreau–Broto autocorrelation, lag k = 0–8; same weights as ATS.
	$ATSC_k w$	Centred Moreau–Broto autocorrelation, lag k = 0–8; weights $w \in \{c, dv, d, s, Z, m, v, se, pe, are, p, i\}$.
	$AATSC_k w$	Average centred Moreau–Broto autocorrelation, lag $k = 0$–8; same weights as $ATSC$.
	$MATS_k w$	Moran autocorrelation index, lag $k = 1$–8; weights $w \in \{c, dv, d, s, Z, m, v, se, pe, are, p, i\}$.
	$GATS_k w$	Geary autocorrelation index, lag k = 1–8; same weights as $MATS$.
**BCUT**	$BCUTw$-$1h$, $BCUTw$-$1l$	Highest and lowest eigenvalues of the Burden matrix weighted by property $w \in \{c, dv, d, s, Z, m, v, se, pe, are, p, i\}$ (24 descriptors total).

Table 4 Matrix Spectral, Connectivity, and E-State Descriptors.

Category	Descriptor(s)	Description
**Matrix Spectral**	$SpAbs\_A$, $SpMax\_A$, $SpDiam\_A$	Spectral absolute value, maximum eigenvalue, and spectral diameter of the adjacency matrix (A).
	$SpAD\_A$, $SpMAD\_A$, $LogEE\_A$	Spectral absolute deviation, mean absolute deviation, and log of Estrada index for A.
	$VE1\_A$–$VE3\_A$, $VR1\_A$–$VR3\_A$	Eigenvector-based and Randić-type spectral coefficients of A.
	$SpAbs\_D$, $SpMax\_D$, ..., $VR3\_D$	Analogous spectral descriptors for the topological distance matrix (D).
	$SpAbs\_Dt$, $SpMax\_Dt$, ..., $VR3\_Dt$, $SM1\_Dt$	Spectral descriptors for the detour matrix (Dt), including $SM1$ (sum of eigenvalues).
	$SpAbs\_Dzw$, ..., $VR3\_Dzw$, $SM1\_Dzw$	Spectral descriptors for property-weighted distance matrices (Dz), $w \in \{Z, m, v, se, pe, are, p, i\}$ (8 matrices, 13 stats each = 104 descriptors).
	SZ, Sm, Sv, Sse, Spe, $Sare$, Sp, Si	Sum of atomic property values over all heavy atoms (Z, m, v, se, pe, are, p, i).
	MZ, Mm, Mv, Mse, Mpe, $Mare$, Mp, Mi	Mean of atomic property values.
**Connectivity**	Xp-kd, Xp-kdv	Path chi indices, orders $k = 0$–7, for σ-electron (d) and valence-electron (dv) weights.
	AXp-kd, AXp-kdv	Average path chi indices, orders $k = 0$–7.
	Xc-kd, Xc-kdv	Cluster chi indices, orders $k = 3$–6.
	Xpc-kd, Xpc-kdv	Path-cluster chi indices, orders $k = 4$–6.
	Xch-kd, Xch-kdv	Chain chi indices, orders $k = 3$–7.
	$Kier1$, $Kier2$, $Kier3$	Kier shape indices (κ_1, κ_2, κ_3).
**E-State**	$NsXY$	Count of atom type XY (e.g., $NsCH3$, $NaaNH$, $NsOH$); covers C, N, O, S, P, Si, Ge, As, Se, Sn, Pb, and halogen environments.
	$SsXY$	E-State index sum over atom type XY.
	$MAXsXY$	Maximum E-State value over atom type XY.
	$MINsXY$	Minimum E-State value over atom type XY.
	$EState\_VSA1$–10	Surface area contributions binned by E-State value.
	$VSA\_EState1$–9	E-State contributions binned by surface area.

Table 5 Surface Area, Ring/Walk, and Information-Theoretic Descriptors.

Category	Descriptor(s)	Description
**Surface Area**	$SlogP\_VSA1$–11	Surface area contributions binned by Wildman–Crippen log P values.
	SMR_VSA1–9	Surface area contributions binned by molar refractivity.
	$PEOE\_VSA1$–13	Surface area contributions binned by Gasteiger partial charges.
**Rings & Walks**	$nRing$, $n3Ring$–$n12Ring$, $nG12Ring$	Total ring count and ring-size counts (3–12 members; $nG12Ring$ for rings > 12).
	$nHRing$, $n3HRing$–$nG12HRing$	Heteroatom-containing ring counts by size.
	$naRing$, $n3aRing$–$nG12aRing$	Aromatic ring counts by size.
	$naHRing$, $n3aHRing$–$nG12aHRing$	Aromatic heteroatom ring counts by size.
	$nARing$, $nAHRing$ and size variants	Aliphatic ring and aliphatic heteroatom ring counts.
	$nFRing$, $nFHRing$, $nFaRing$, $nFaHRing$, $nFARing$, $nFAHRing$ and size variants	Fused-ring counts (fused hetero, aromatic, aliphatic variations) by ring size.
	$MWC01$–$MWC10$, $TMWC10$	Molecular Walk Counts of lengths 1–10 and total.
	$SRW02$–$SRW10$, $TSRW10$	Self-Returning Walk counts of lengths 2–10 and total.
**Information**	$IC0$–$IC5$	Information content indices at graph distance 0–5.
	$TIC0$–$TIC5$	Total information content indices.
	$SIC0$–$SIC5$	Structural information content indices.
	$BIC0$–$BIC5$	Bonding information content indices.
	$CIC0$–$CIC5$	Complementary information content indices.
	$MIC0$–$MIC5$	Modified information content indices.
	$ZMIC0$–$ZMIC5$	Atomic number-weighted modified information content.
**Distance Codes**	MID, $AMID$	Mean information-theoretic distance and its average.
	MID_h, $AMID\_h$	MID / $AMID$ restricted to hydrogen-containing paths.
	MID_C, $AMID\_C$; MID_N, $AMID\_N$; MID_O, $AMID\_O$; MID_X, $AMID\_X$	Element-resolved mean distances (C, N, O, halogen).

Table 6 Topological Charge, Walk/Path Counts, and Topochemical (ETA) Descriptors.

Category	Descriptor(s)	Description
**Topological Charge**	$GGI1$–$GGI10$	Galvez topological charge indices of order 1–10.
	$JGI1$–$JGI10$	Mean topological charge indices of order 1–10.
	$JGT10$	Total topological charge index (sum over orders 1–10).
**Path/Walk Counts**	$MPC2$–$MPC10$, $TMPC10$	Molecular path counts of length 2–10 and total.
	$piPC1$–$piPC10$, $TpiPC10$	π-path counts of length 1–10 and total.
	$MDEC\text{-}ij$	Molecular distance-edge carbon descriptors ($ij \in \{11, 12, 13, 14, 22, 23, 24, 33, 34, 44\}$).
	$MDEO\text{-}ij$	Molecular distance-edge oxygen descriptors ($ij \in \{11, 12, 22\}$).
	$MDEN\text{-}ij$	Molecular distance-edge nitrogen descriptors ($ij \in \{11, 12, 13, 22, 23, 33\}$).
**Topochemical (ETA)**	ETA_alpha, $AETA\_alpha$	ETA alpha (composite atom contribution) and average.
	ETA_shape_p, ETA_shape_y, ETA_shape_x	ETA shape descriptors (parabolic, Y-shaped, X-shaped molecular profiles).
	ETA_beta, $AETA\_beta$, ETA_beta_s, $AETA\_beta\_s$	ETA beta (total and sigma-only) and averages.
	ETA_beta_ns, $AETA\_beta\_ns$, $ETA\_beta\_ns\_d$, $AETA\_beta\_ns\_d$	ETA beta non-sigma contributions and their differences.
	ETA_eta, $AETA\_eta$, ETA_eta_L, $AETA\_eta\_L$	ETA eta indices (local and average).
	ETA_eta_R, $AETA\_eta\_R$, ETA_eta_RL, $AETA\_eta\_RL$	ETA eta R-branch indices and averages.
	ETA_eta_F, $AETA\_eta\_F$, ETA_eta_FL, $AETA\_eta\_FL$	ETA eta F indices and averages.
	ETA_eta_B, $AETA\_eta\_B$, ETA_eta_BR, $AETA\_eta\_BR$	ETA eta B indices and averages.
	ETA_dAlpha_A, ETA_dAlpha_B	Delta-alpha ETA descriptors (A and B variants).
	$ETA\_epsilon\_1$–5	ETA epsilon descriptors (five variants).
	$ETA\_dEpsilon\_A$–D	Delta-epsilon ETA descriptors.
	ETA_dBeta, $AETA\_dBeta$	Delta-beta and average delta-beta.
	ETA_psi_1, ETA_dPsi_A, ETA_dPsi_B	ETA psi and delta-psi descriptors.

3 Details on MolFuSE Workflow

MolFuSE workflow starts with the user selecting the "molecular function" from a pull down menu. This menu is populated from UniProt's keyword Identifier (i.e., ID line code) that are within the molecular functions category (e.g., "ID Transferase). Consequently, all proteins that are tagged with the associated Accession (i.e., AC line code) value (e.g., AC KW-0808 for Transferase) are then pooled and the resulting ligands are included in the similarity search. For each evaluated target T, we created a *target-specific reference set* based on the selected keyword and by explicitly removing all compounds known to bind target T. Thus, $\mathrm{RS}_T = \mathrm{RS}_{\mathrm{class}} \setminus \mathrm{Actives}_T$. This ensured zero data leakage for every individual evaluation target.

References

Banerjee A, Kar S, Roy K, et al (2025) From feature-based chemical similarity to chemical language models–a paradigm shift in computer-aided molecular design and property predictions. WIREs Comput Mol Sci 15(6):e70057. URL https://doi.org/10.1002/wcms.70057

Becht E, McInnes L, Healy J, et al (2019) Dimensionality reduction for visualizing single-cell data using umap. Nature biotechnology 37(1):38–44

Chithrananda S, Grand G, Ramsundar B (2020) Chemberta: large-scale self-supervised pretraining for molecular property prediction. arXiv preprint arXiv:201009885

Cihan Sorkun M, Mullaj D, Koelman JVA, et al (2022) Chemplot, a python library for chemical space visualization. Chemistry-Methods 2(7):e202200005

Cleves AE, Jain AN (2020) Structure-and ligand-based virtual screening on dude+: performance dependence on approximations to the binding pocket. Journal of chemical information and modeling 60(9):4296–4310

Cleves AE, Johnson SR, Jain AN (2019) Electrostatic-field and surface-shape similarity for virtual screening and pose prediction. Journal of computer-aided molecular design 33(10):865–886

Fabian B, Edlich T, Gaspar H, et al (2020) Molecular representation learning with language models and domain-relevant auxiliary tasks. arXiv preprint arXiv:201113230

Guo Q, Hernandez-Hernandez S, Ballester PJ (2024) Scaffold splits overestimate virtual screening performance. In: Artificial Neural Networks and Machine Learning - ICANN 2024. Springer Nature Switzerland, Cham, pp 58–72

Guo Q, Hernandez-Hernandez S, Ballester PJ (2025) Umap-based clustering split for rigorous evaluation of ai models for virtual screening on cancer cell lines*. Journal of Cheminformatics 17(1):94. URL https://doi.org/10.1186/s13321-025-01039-8

Hernández-Hernández S, Ballester PJ (2023) On the best way to cluster nci-60 molecules

Kobak D, Berens P (2019) The art of using t-sne for single-cell transcriptomics. Nature communications 10(1):5416

Li J, Liu W, Song Y, et al (2020) Improved method of structure-based virtual screening based on ensemble learning. RSC advances 10(13):7609–7618

Liu Y, Moretti R, Wang Y, et al (2025) Advancements in ligand-based virtual screening through the synergistic integration of graph neural networks and expert-crafted descriptors. Journal of Chemical Information and Modeling 65(10):4898–4905

Lovrić M, DJuričić T, Tran HT, et al (2021) Should we embed in chemistry? a comparison of unsupervised transfer learning with pca, umap, and vae on molecular fingerprints. Pharmaceuticals 14(8):758

Van der Maaten L, Hinton G (2008) Visualizing data using t-sne. Journal of machine learning research 9(11)

McGaughey GB, Sheridan RP, Bayly CI, et al (2007) Comparison of topological, shape, and docking methods in virtual screening. Journal of chemical information and modeling 47(4):1504–1519

McInnes L, Healy J, Saul N, et al (2018) Umap: Uniform manifold approximation and projection. Journal of Open Source Software 3(29)

Mswahili ME, Jeong YS (2024) Transformer-based models for chemical smiles representation: A comprehensive literature review. Heliyon 10(20). https://doi.org/10.1016/j.heliyon.2024.e39038, URL https://doi.org/10.1016/j.heliyon.2024.e39038

Mysinger MM, Carchia M, Irwin JJ, et al (2012) Directory of useful decoys, enhanced (dud-e): better ligands and decoys for better benchmarking. Journal of medicinal chemistry 55(14):6582–6594

Nguyen LH, Holmes S (2019) Ten quick tips for effective dimensionality reduction. PLOS Computational Biology 15(6):e1006907. https://doi.org/10.1371/journal.pcbi.1006907, URL https://doi.org/10.1371/journal.pcbi.1006907

Oprea TI, Gottfries J (2001) Chemography: the art of navigating in chemical space. Journal of combinatorial chemistry 3(2):157–166

Orlov AA, Akhmetshin TN, Horvath D, et al (2025) From high dimensions to human insight: Exploring dimensionality reduction for chemical space visualization. Molecular Informatics 44(1):e202400265

Probst D, Reymond JL (2020) Visualization of very large high-dimensional data sets as minimum spanning trees. Journal of Cheminformatics 12(1):12

Rohrer SG, Baumann K (2009) Maximum unbiased validation (muv) data sets for virtual screening based on pubchem bioactivity data. Journal of chemical information and modeling 49(2):169–184

Sciabola S, Torella R, Nagata A, et al (2022) Critical assessment of state-of-the-art ligand-based virtual screening methods. Mol Inf 41(11):2200103. URL https://doi.org/10.1002/minf.202200103

Tran-Nguyen VK, Jacquemard C, Rognan D (2020) Lit-pcba: an unbiased data set for machine learning and virtual screening. Journal of chemical information and modeling 60(9):4263–4273

Umer MS, Nabeel M, Athar U, et al (2025) Large language models meet molecules: A systematic review of advances and challenges in ai-driven cheminformatics. Archives of Computational Methods in Engineering URL https://doi.org/10.1007/s11831-025-10437-y

Vogel SM, Bauer MR, Boeckler FM (2011) Dekois: Demanding evaluation kits for objective in silico screening: A versatile tool for benchmarking docking programs and scoring functions. Journal of chemical information and modeling 51(10):2650–2665

Yasuo N, Sekijima M (2019) Improved method of structure-based virtual screening via interaction-energy-based learning. Journal of chemical information and modeling 59(3):1050–1061
